# Insights into RNA‐mediated pathology in new mouse models of Huntington's disease

**DOI:** 10.1096/fj.202401465R

**Published:** 2024-11-27

**Authors:** Magdalena Wozna‐Wysocka, Magdalena Jazurek‐Ciesiolka, Lukasz Przybyl, Dorota Wronka, Julia Oliwia Misiorek, Joanna Suszynska‐Zajczyk, Grzegorz Figura, Adam Ciesiolka, Paula Sobieszczanska, Anna Zeller, Magdalena Niemira, Pawel Michal Switonski, Agnieszka Fiszer

**Affiliations:** ^1^ Institute of Bioorganic Chemistry Polish Academy of Sciences Poznan Poland; ^2^ Department of Biochemistry and Biotechnology Poznan University of Life Sciences Poznan Poland; ^3^ Genomics and Epigenomics Laboratory, Clinical Research Centre Medical University of Bialystok Bialystok Poland; ^4^ Present address: Department of Bioenergetics, Institute of Molecular Biology and Biotechnology Adam Mickiewicz University Poznan Poland; ^5^ Present address: Department of Gene Expression, Institute of Molecular Biology and Biotechnology Adam Mickiewicz University Poznan Poland

**Keywords:** Huntington's disease, mouse model, neurodegenerative diseases, polyglutamine disorders, RNA toxicity

## Abstract

Huntington's disease (HD) is a neurodegenerative polyglutamine (polyQ) disease resulting from the expansion of CAG repeats located in the ORF of the huntingtin gene (*HTT*). The extent to which mutant mRNA‐driven disruptions contribute to HD pathogenesis, particularly in comparison to the dominant mechanisms related to the gain‐of‐function effects of the mutant polyQ protein, is still debatable. To evaluate this contribution in vivo, we generated two mouse models through a knock‐in strategy at the *Rosa26* locus. These models expressed distinct variants of human mutant *HTT* cDNA fragment: a translated variant (HD/100Q model, serving as a reference) and a nontranslated variant (HD/100CAG model). The cohorts of animals were subjected to a broad spectrum of molecular, behavioral, and cognitive analysis for 21 months. Behavioral testing revealed alterations in both models, with the HD/100Q model exhibiting late disease phenotype. The rotarod, static rod, and open‐field tests showed some motor deficits in HD/100CAG and HD/100Q model mice during the light phase, while ActiMot indicated hyperkinesis during the dark phase. Both models also exhibited certain gene deregulations in the striatum that are related to disrupted pathways and phenotype alterations observed in HD. In conclusion, we provide in vivo evidence for a minor contributory role of mutant RNA in HD pathogenesis. The separated effects resulting from the presence of mutant RNA in the HD/100CAG model led to less severe but, to some extent, similar types of impairments as in the HD/100Q model. Increased anxiety was one of the most substantial effects caused by mutant *HTT* RNA.

## Graphical abstract

The behavioral and molecular characterization was performed for Huntington's disease mouse models expressing a fragment of the human *HTT* gene, with mutation of ~100 CAG repeats, in the nontranslated version (HD/100CAG) and the translated version (HD/100Q).

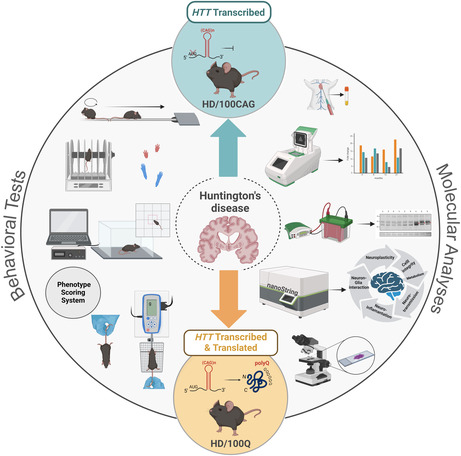

**Abbreviations**
AKT2: AKT serine/threonine kinase 2
DEGs: differentially expressed genes
EphA7: EPH receptor A7
FC: fold change
HA: hemagglutinin
HD: Huntington's disease
HTT: huntingtin
IGF1R: insulin growth factor 1 receptor
INSR: insulin receptor
IPA: ingenuity pathway analysis
MCC: maximal clique centrality
MEFs: mouse embryonic fibroblasts
mHTT: mutant huntingtin
MSNs: medium spiny neurons
PARP1: poly(ADP‐ribose) polymerase 1
polyQ: polyglutamine
PPI: protein–protein interaction
RAN: repeat‐associated non‐ATG translation
RF: representation factor
smFISH: single‐molecule fluorescent in situ hybridization
SRSF4: serine and arginine‐rich splicing factor 4
WT: wild‐type

## 1 INTRODUCTION

Huntington's disease (HD) is a progressive and fatal inherited neurodegenerative disorder caused by an increase in the number of trinucleotide CAG repeats in exon 1 of the huntingtin gene (*HTT*), which is located on the short arm of chromosome 4.^1^, ^2^ This autosomal dominant mutation results in a long stretch of glutamine (Q) residues in the huntingtin protein (HTT). The normal number of CAG repeats in *HTT* is typically approximately 17, whereas a CAG repeat length of 36 is related to pathology. Individuals with 36–39 CAG repeats in *HTT* have an increased risk of developing the disease, while a repeat number above 39 inevitably leads to HD symptoms. The onset of HD occurs at a mean age of ~45 years.^3^ However, the most severe juvenile form of HD, associated with more than 60 CAG repeats in *HTT*, emerges before 20 years of age.^4^, ^5^ Brain areas responsible for cognition, personality, and movement are particularly affected in HD, leading to the classic triad of symptoms: cognitive deficits (difficulties with concentration and emotion recognition), behavioral issues (neuropsychiatric symptoms such as apathy), and motor abnormalities (often marked by chorea and dystonia).^6^ Striatal medium spiny neurons (MSNs) are among the most vulnerable neuronal populations in the degenerating brain; however, as the disease progresses, neuronal dysfunction and prominent atrophy also occur in the cerebral cortex, subcortical white matter, thalamus, and hypothalamic nuclei.^7^, ^8^, ^9^, ^10^ While HD is recognized as a brain disorder, it also has substantial effects in the periphery.^11^, ^12^, ^13^

Although the pathogenic *HTT* mutation was discovered in 1993,^2^ the precise mechanisms of neurodegeneration in HD remain elusive.^14^ The contribution of mutant HTT (mHTT) to HD pathogenesis through a protein gain‐of‐function mechanism has been extensively studied. Numerous studies have shown that an abnormally elongated polyQ tract induces conformational changes in mHTT that alter protein–protein interactions (PPIs) and stimulate aggregate formation.^14^, ^15^, ^16^ As a result of altered mHTT functioning, many fundamental cellular processes are affected, ultimately leading to neuronal dysfunction and death.^6^, ^17^ mHTT has been associated with transcriptional dysregulation, altered energy homeostasis, mitochondrial dysfunction, oxidative stress, compromised autophagy, impaired cell transport, synaptic dysfunction, altered neurotransmitter signaling, neurotrophic deficits, and inflammation.^3^, ^18^ Transcriptional dysregulation is regarded as an initial alteration in HD and is correlated with brain atrophy, predominantly in the caudate nucleus, as well as with pathology observed in peripheral tissues.^19^, ^20^, ^21^, ^22^

The contribution of *HTT* RNA toxicity to HD is not fully understood. Growing evidence indicates that expanded CAG repeats can induce toxicity at the RNA level and there are several postulated mechanisms of RNA‐mediated pathology in HD. Transcribed CAG repeats cause abnormal interactions between mutant RNA and proteins, which results in deregulated splicing of *mHTT* RNA itself and other transcripts, the formation of intranuclear foci/clusters, and the generation of short CAG‐containing RNAs.^23^, ^24^, ^25^, ^26^, ^27^, ^28^, ^29^, ^30^, ^31^, ^32^ Furthermore, *mHTT* RNA also serves as a template for repeat‐associated non‐ATG (RAN) translation. This unconventional protein translation mechanism results in the synthesis of unusual proteins, which have been detected in the postmortem brains of HD patients, especially in the striatum.^33^ However, the role of RAN–translated proteins in HD is still unclear. Overall, determining the extent to which disruption of mutant RNA‐mediated pathways contributes to the pathogenesis of HD is extremely difficult, given the coexistence of mutant mRNA and protein factors. Thus, additional models are needed that enable separate analysis of toxic effects of the mutant protein and RNA.

To date, more than 20 different mouse models of HD recapitulating distinct disease‐related pathological, functional, and behavioral phenotypes have been generated. The characteristics of each model depend on the genetic strategy; for example, there are models expressing truncated human *HTT* (encoding N‐terminal fragments) and full‐length human mutant *HTT*, including the humanized form of the gene and a large group of knock‐in models with expanded CAG repeats inserted into mouse *Htt* exon 1 (reviewed in Ref. [34]). HD mouse models have many advantages in mimicking some of the behavioral and neuropathological features observed in patients but the mechanisms potentially involved in HD pathogenesis still require addressing.

**FIGURE 1:**
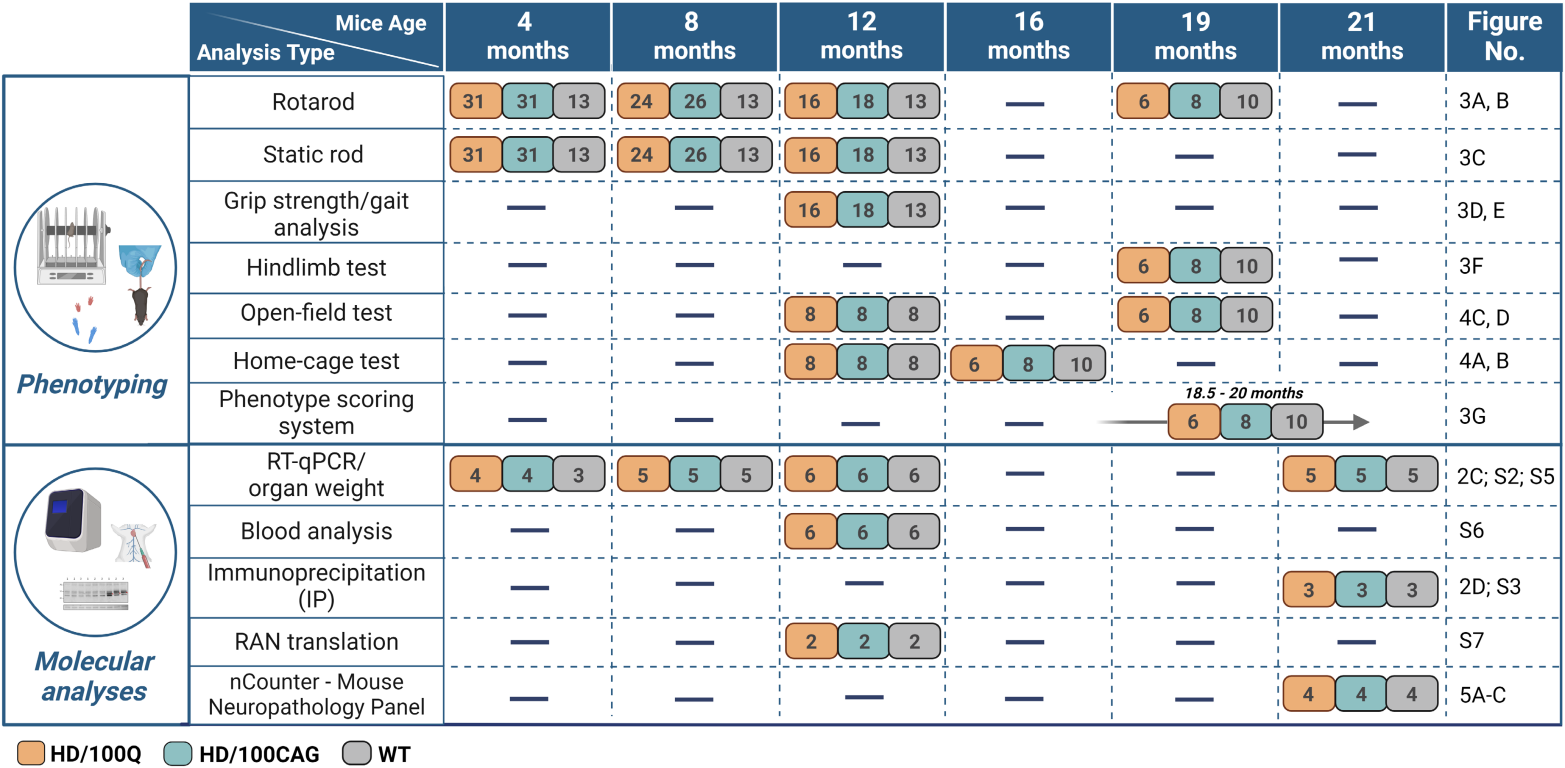
Summary of the experiments conducted at different timepoints. The number of animals in each group is given in rounded squares; the groups corresponding to the colors are shown below the table. In the last column, specific panels of figures are indicated where the results of respective experiments are presented.

This study aimed to assess the contribution of mutant RNA to HD pathogenesis based on in vivo observations in mouse models. To allow direct comparison, we generated two models that each harbor the same mutant *HTT* transgene but differ in its ability to be translated. One of the models expresses the transgene, with approximately 100 CAG repeats, only at the RNA level, and the other expresses the transgene at both the RNA and protein levels. We performed a set of molecular, motor, and behavioral tests (Figure 1) to test the hypothesis that the *mHTT* transcript itself has pathological implications for the development and progression of HD.

## 2 MATERIALS AND METHODS

### 2.1 Generation of HD/100CAG and HD/100Q transgenic mice

#### 2.1.1 Vector design

A fragment of human *HTT* cDNA containing exons 1 to 3 and some of exon 4 (up to glutamate E169) (Text S1), followed by the coding sequence of the HA tag and a linker sequence, was generated via gene synthesis (GenScript). The synthesized gene contained 98 CAG repeats (a tract encoding 100Q repeats due to a CAA codon preceding the last CAG codon) with either two ATG codons (for the HD/100Q model) or the termination codon TGA (for HD/100CAG) at positions M1 and M8. The *HTT* fragment and MS2 aptamer obtained from the phage‐cmv‐cfp‐24xms2 (Addgene #40651) vector were first backcloned into the pCAGEN (Addgene #11160) plasmid and then into the D222 vector at the XhoI and NotI restriction sites and subsequently transferred to Rosa26.V targeting plasmid using the BssHII/AgeI sites of the D222 vector and the AscI/XmaI sites of the Rosa26.V vector. The final targeting plasmid contained Rosa26 homology arms, a CMV early enhancer/chicken beta actin (CAG) promoter upstream of the coding sequence, and a bovine growth hormone polyA site downstream of the MS2 aptamer. To prevent potential embryonic lethality and increase the versatility of the models, a neo/STOP cassette flanked by loxP sites was introduced between the CAG promoter and the *HTT* coding sequence.

#### 2.1.2 Clonal selection

The HD/100CAG and HD/100Q transgenic models were generated by PolyGene AG. Fifteen milligrams of the targeting plasmid was linearized with SacI and electroporated into 6 × 10^6^ C57Bl/6‐derived ES cells. G418 (0.2 mg/mL) selection was performed for 8 days to ensure stable transfection. Genomic DNA from isolated clones was digested with BglII and hybridized to a Rosa26 probe generated by the Rosa_forward and Rosa_reverse primers. Subsequently, positive clones were tested by long‐range PCR to confirm correct homologous recombination on the long homology arm using the C011.5 and E573.3 primers. To confirm correct homologous recombination with the Rosa26 locus, two additional Southern blot analyses were performed, one with the same 5′ external probe as before (Rosa26 probe) and BclI‐digested DNA and the second with a 3′ external probe and ApaI‐digested DNA. The second probe was generated by the RO26.6 and RO26.7 primers. See Text S2 for more details.

#### 2.1.3 Breeding

Following the injection of blastocysts, the surviving blastocysts were transferred into foster mice of the CD‐1 strain. The resulting chimeric animals were then bred with C57Bl/6N Flp‐deleter mice to generate either CAG‐driven or Rosa‐driven transgene expression. However, the latter approach was discontinued thereafter. To determine whether the CAG promoter was present in the target allele, the CAG.1 and LRPCRneo1 primers were used for screening. F1 mice that tested positive for the CAG promoter were backcrossed twice with C57Bl/6N mice. Subsequently, they were bred with Cre transgenic mice to eliminate the neo/STOP cassette and achieve constitutive activation of the CAG promoter. The primers used for the generation and verification of HD/100Q and HD/100CAG transgenic mice are listed in Table S1.

### 2.2 Experimental animals and husbandry

Male homozygous transgenic mice (HD/100Q and HD/100CAG) and wild‐type (WT) littermates aged between 4 and 21 months were used in all experiments. The animals were housed in the animal facility of the Center for Advanced Technologies Adam Mickiewicz University in Poznan, Poland (CAT AMU), at a controlled temperature and humidity under specific pathogen‐free (SPF) conditions on a 12‐h light/12‐h dark cycle and provided access to water and food ad libitum. All experiments were performed with the approval of the Local Ethical Committee for Animal Experiments in Poznan, Poland (no. 47/2018; approved on 28.11.2018, with later annexes).

### 2.3 Genotyping

DNA was extracted from mouse tails using an EZ‐10 Spin Column Genomic DNA Minipreps Kit (Bio Basic) according to the manufacturer's instructions. The extracted DNA was used for genotyping by PCR using GoTaq G2 Flexi DNA Polymerase (Promega). Different sets of primers were used to genotype mice that were heterozygous or homozygous for the transgene, that expressed or did not express the STOP cassette, or that expressed or did not express the Cre recombinase gene (see Table S1). The following PCR conditions were used: 1 cycle of 95°C/5 min; 35 cycles of 95°C/30 s, 62°C*/30 s, 72°C/45 s; 1 cycle of 72°C/7 min. *The annealing temperature was different depending on the set of primers used. All reactions were performed on a Bio‐Rad T100 thermocycler. The PCR products were separated on 1.5% agarose gels in TBE buffer and stained with ethidium bromide (Sigma).

### 2.4 Capillary electrophoresis of PCR products for CAG repeat number quantitation

Total genomic DNA isolated from mouse tails (used for genotyping) was used as a template. *HTT* transgene fragments containing repeat tracts were amplified via PCR with Phusion Flash High‐Fidelity PCR Master Mix (Thermo Fisher Scientific) and labeled primers (Table S1). The PCR mixture contained 0.63 μM of each primer and 100 ng of genomic DNA in a 50 μL reaction volume. The program for amplification was as follows: 98°C for 1 min; 11 cycles of 98°C for 15 s and 72°C for 15 s; 21 cycles of 98°C for 15 s, 62°C for 15 s, and 72°C for 15 s; and 72°C for 5 min, using thermocycler as mentioned above. The PCR products obtained prior to capillary electrophoresis were cleaned using a Syngen Gel/PCR ME Mini Kit. To determine the CAG repeat number in *HTT* transgene, we used an allelic ladder composed of the PCR products obtained from genomic DNA from four HD patients with *HTT* alleles containing known numbers of CAG repeats: 18/45, 15/85, 17/71, and 12/62. Capillary electrophoresis was performed at the Laboratory of Molecular Biology Techniques (University of Adam Mickiewicz in Poznan). Briefly, 1 μL of the sample was added to 9 μL of formamide (Applied Biosystems) containing 0.25 μL of GeneScan™ 600 LIZ™ Size Standard. Next, the samples were denatured at 95°C for 5 min and cooled to 10°C. Capillary electrophoresis was performed on an ABI Prism 3130xl instrument with 36 cm capillaries, POP‐7™ polymer, and a G5 filter according to the manufacturer's protocol. The results were analyzed with Peak Scanner Software 1.0.

### 2.5 Tissue and blood sample collection

Tissues were collected at four timepoints, that is, 4, 8, 12, and 21 months of age. The animals were euthanized by an overdose of isoflurane (Isotek, LABORATORIOS KARIZOO), and the mice and their organs were subsequently weighed. Next, the hearts, spleens, and left kidneys were immediately snap‐frozen in liquid nitrogen, and the brains were chilled briefly on ice in PBS/HBSS, and different brain regions, including the right and left cortex, striatum, hippocampus, midbrain, and cerebellum, were microdissected.

Blood samples were collected from 12‐month‐old mice (*n* = 6 per group) immediately after the animals were sacrificed by standard cardiac puncture using a 1 mL syringe and a 29‐gauge needle. The samples were allowed to completely clot on the benchtop for 30 min. The serum was separated by centrifugation at 1300× *g* for 15 min at room temperature and then stored at −80°C until further analysis.

### 2.6 Blood and urine sample analysis

The concentrations of alanine aminotransferase, cholesterol, alkaline phosphatase, and uric acid were measured using a Mindray BS‐120 automated clinical chemistry analyzer and ACCENT‐200 biochemical reagents following the manufacturer's instructions. Urinal total homocysteine (tHcy) was assayed by an HPLC‐based method with post‐column derivatization as previously described.^35^

### 2.7 RNA extraction and RT–qPCR

Tissues were homogenized in TRIzol reagent (Thermo Fisher Scientific) by being passed through a syringe and needle at least 7–10 times until a homogeneous lysate was obtained. Total RNA was isolated from right striatal, cortical, and hippocampal tissue using a total RNA Zol‐Out D kit (A&A Biotechnology) and subsequently treated with DNase I according to the manufacturer's protocol. The RNA concentration and purity were determined using a DeNovix DS‐11 spectrophotometer (DeNovix Inc.). To assess RNA integrity, 500 ng of total RNA per sample was mixed with loading dye, subjected to electrophoresis in 1% agarose in TAE buffer, and stained with ethidium bromide. Good‐quality samples were identified by visualization of intact 28S and 18S ribosomal RNA and the absence of smearing.

Total RNA (500 ng) was reverse transcribed into cDNA using a High‐Capacity cDNA Reverse Transcription Kit with RNase inhibitor (Thermo Fisher Scientific) in a total volume of 20 μL, according to the manufacturer's protocol. The resulting cDNA was diluted 10‐fold with RNase‐free water for qPCR analysis. Gene expression analysis was performed on a CFX Connect Real‐Time PCR Detection System (Bio‐Rad) using SsoAdvanced Universal SYBR Green Supermix (Bio‐Rad) in a 10 μL reaction under the following conditions: preheating at 95°C for 30 s followed by 40 cycles of denaturation at 95°C for 15 s and annealing at 60°C for 30 s. The relative expression of the human huntingtin gene (*HTT*, transgene) and endogenous mouse huntingtin gene (*Htt*) was normalized to the mean expression of two endogenous reference genes, ATP synthase F1 subunit beta (*Atp5b*) and glyceraldehyde‐3‐phosphate dehydrogenase (*Gapdh*), which was stable regardless of the genotype and type of tissue (see primers in Table S1). Finally, the expression of endogenous *Htt* in the striatum, cortex, and hippocampus of HD/100Q and HD/100CAG mice of different ages was normalized to that in 4‐month‐old WT mice, while the *HTT* transgene expression in HD/100CAG mice was normalized to that in 4‐month‐old HD/100Q mice. Gene expression was calculated using the 2^(−ΔΔCT) method.

### 2.8 Immunoprecipitation

Frozen left cortical tissues from 21‐month‐old mice were lysed in PB buffer (60 mM Tris‐base, 2% SDS, 10% sucrose, and fresh 2 mM PMSF) supplemented with Halt Protease and Phosphatase Inhibitor Cocktail (Thermo Fisher Scientific) for 30 min on ice and sonicated three times (30 s on and 30 s off) (Bioruptor, Diagenode). IP was performed on 500 μg of total lysate by incubation with Anti‐HA Agarose (Pierce) for 16 h at 4°C with rotation, according to the manufacturer's protocol. After centrifugation and two washes with TBS, proteins were subsequently eluted from the resin with sample buffer, resolved by 10% SDS/PAGE, and transferred overnight to nitrocellulose membranes (Amersham). The membranes were stained with Ponceau S for verification of consistency in sample preparation and blocked with 5% non‐fat dry milk in TBS‐0.1% Tween‐20 for 1 h. The immunoprecipitated HTT fragment was detected by immunoblotting with a rabbit anti‐HA antibody (Cell Signaling Technology; 1:1000) overnight at 4°C. Then, the membrane was incubated with an HRP‐conjugated anti‐rabbit secondary antibody (Jackson ImmunoResearch) diluted 1:1000 for 1 h at RT. The immunoreactive bands were detected using WesternBright Quantum HRP Substrate (Advansta) and a G:BOX chemiluminescence camera (Syngene).

### 2.9 MEFs isolation and culture

Mouse embryonic fibroblasts (MEFs) were obtained from HD/100CAG and WT mice. Embryos were harvested from the isolated uterine horns of pregnant female mice at E13.5. The embryos were then placed on a culture plate containing PBS. The head and viscera were removed, and the remaining tissue was washed with fresh PBS. Subsequently, the tissue was minced using a pair of scissors, pooled, and digested with 1 mL of 0.25% trypsin (Gibco) at 37°C for 10 min. Trypsinization was stopped with 5 mL of DMEM (Gibco) supplemented with 10% FBS (Biowest) and 1× Pen/Strep (Gibco), and the tissue was pipetted 20 times. The cells were transferred to a 10 cm culture dish containing 14 mL of DMEM supplemented with 10% FBS and 1× Pen/Strep and incubated overnight at 37°C in 5% CO_2_. Next, the medium was replaced with fresh medium, and the MEFs were incubated until they reached confluence. At this point, the cells were frozen in DMEM supplemented with 10% FBS and 10% DMSO.

### 2.10 smFISH

Probe, buffers, and a protocol from Stellaris RNA FISH Technology (Biosearch Technologies) were used for the detection of MS2‐tagged *HTT* mRNA. Briefly, HD/100CAG MEFs and WT MEFs were seeded on an 18 mm cover glass in a 12‐well cell culture plate and grown for at least 24 h in DMEM supplemented with 10% FBS and 1× Pen/Strep. The cells were then washed with PBS, fixed in 4% paraformaldehyde/PBS for 30 min at RT, permeabilized in 0.5% Triton X‐100 (Sigma–Aldrich) supplemented with 2 mM vanadyl (New England BioLabs) and prehybridized in Wash Buffer A supplemented with 10% formamide for 5 min at RT. Hybridization with 125 nM Quasar670‐conjugated probes (5′ and 3′‐labeled, targeting the MS2 region, from Yunger et al.^36^, Nature Protocols) was performed in Stellaris Hybridization Buffer supplemented with 10% formamide, followed by incubation at 37°C overnight. Washing was performed with Wash Buffer A for 30 min at 37°C and then with Wash Buffer B for 5 min at RT. SlowFade Diamond Antifade Mountant (Invitrogen) was used for nuclear staining. Images were captured with a Leica DMI6000 inverted fluorescence microscope equipped with a DFC360 FX camera. The Leica A excitation/emission filter set was used for DAPI and the Chroma 49009 excitation/emission filter set was used for Quasar670.

### 2.11 Behavioral testing

Behavioral tests were carried out by investigators who were blinded to the genotypes of the animals. The tests were performed after acclimatization and handling. All tests were performed at the same time each day, starting in the morning, in a specialized room dedicated for behavioral testing.

#### 2.11.1 Rotarod test

The rotarod test was performed using 47750 Rota‐Rod NG instrument (Ugo‐Basile) and used to assess the coordination, motor skills, and learning capabilities of the mice. The test consisted of three trials per day with 1‐h intersession intervals and was carried out for 3 days. The mice were tested in acceleration mode; the initial speed was 3 rpm, and a maximum speed of 40 rpm was reached after 300 s. Training trials were performed on the first and second days to familiarize the animals with the equipment, and the test trial was performed on the third day. On the first 2 days, the trials lasted a maximum of 300 s, after which if the mouse had not fallen from the rod, it was removed and placed in its home cage. On the third day, the test continued until the animal fell off the rod or achieved 600 s.

First, the mouse was removed from its home cage and placed on a rod rotating at a speed of 3 rpm facing against the direction of rotation. Once the animal was correctly placed on the rod, the test began. Each mouse was allowed to spin around on the rod without making any movements, clinging on the rod without falling for three times, if a mouse made no attempt to walk, it was removed from the device and returned to its home cage.

Three parameters were measured during the test: (I) the latency, that is, the time the mouse spent on the rod during one trial [s]; (II) the speed, the speed at which the rod was rotating when the animal fell off the rod [rpm]; and (III) the distance, that is, the distance that the mouse had traveled when it fell off the rod [cm].

#### 2.11.2 Static rod test

The static rod test was used to measure locomotor performance and balance. The device used for this test consisted of a 60‐cm‐long rod with a 17 mm diameter that was horizontally attached on one side to a rack placed 1 m above the floor. Soft material was placed under the rod to protect the mouse when it fell from the rod. Each animal was required to turn around at the free end of the rod and reach safety at the other end of the rod, where bedding and feed were present. During the test, two parameters were measured: the time required to turn around and the time required to traverse the rod. The test was performed for 2 days; on the first day, the animals were trained and familiarized with the equipment, and the actual test was performed on the second day. The test was repeated at 4, 8, and 12 months of age.

#### 2.11.3 Hindlimb test

Hind limb clasping was evaluated as previously described.^37^, ^38^ Briefly, the mice were held by the tail, and the hind limb clasping was observed for 10 s. A score of 0 indicated that the hind limbs were set far from the abdomen and that the toes were wide open. One point was awarded if one or both limbs partially moved toward the abdomen but did not touch it and the toes were wide open. Two points were given if both hind limbs partially moved toward and touched the abdomen but did not touch each other. If both limbs were brought close to the abdomen and intertwined, three points were awarded. Each mouse was evaluated at least two times per day, and the results were averaged. The hind limb clasping test was performed at 19 months of age.

#### 2.11.4 Gait analysis

The test is used to detect gait abnormalities among the animals. During the test, each mouse's paws were painted with nontoxic paint (front paws—red, hind paws—blue), and the animal was placed in a tunnel measuring 57 cm × 8.2 cm (length × width) lined with a strip of Whatman tissue paper of the same dimensions. The mouse walked through the tunnel, leaving paw prints on the tissue paper. Each test consisted of three passes. For each strip of paper, we measured the walking width (the distance between lines drawn from the center of the paw prints), the stride length (the distance between the center of the hind paws), and interlimb coordination (the overlap between the hind and front paws). Three replicate measurements were averaged for each parameter. The test was performed when the mice were 12 months of age.

#### 2.11.5 Grip strength

The grip strength of 12‐month‐old mice was measured with a homemade device. The mice were removed from their cages and placed on a grid connected to a sensor, which was used to measure the force with which each mouse pulled the grid in the opposite direction. Each animal was held by the tail and dragged across the grid in such a way that it was able to grab individual bars. The procedure was repeated five times, and the average of five replicate measurements was calculated; the results are presented as the force in grams.

#### 2.11.6 Phenotype scoring system

We used a composite phenotype scoring system to assess the phenotype of the animals as previously described.^37^ Briefly, four parameters were assessed: coordination (the ledge test), muscle alignment (the hind limb clasping test), gait, and increased curve of the spine (kyphosis). Every animal was assessed twice in 1 day, and the performance of the mice in each test was scored on a 0–3 scale; 0 indicated the absence of a phenotype and 3 indicated the presence of a conspicuous phenotype. The tests were repeated every 2 weeks from 18.5 to 20 months of age and carried out by two independent experimenters. The scores awarded during each repetition test were averaged, and the final result for each timepoint is the sum of the average scores.

#### 2.11.7 Home cage activity test

Home cage activity was monitored using the ActiMot2 system (TSE Systems) throughout the circadian cycle. The mice were placed in conventional cages equipped with bedding and ad libitum food and water. Measurements were carried out for 22 h (10 h in the light phase and 12 h in the dark phase), and 1‐min recordings were collected using PhenoMaster V4.8.9 software. Recordings from the first hour of the test were excluded from the analysis to allow acclimatization. Six parameters were measured: general activity, that is, the sum of ambulatory and fine movements; activity in the center and periphery; the distance traveled in 1 min; the total distance traveled; and the total number of vertical movements. Fine movements were defined as repeated breaks of the same light beam. Ambulatory movements were defined as breaks of adjacent beams by the mouse movements in the cage as previously described.^39^ The test was performed at 12 and 16 months of age.

#### 2.11.8 Open‐field test

The open‐field test, a sensorimotor test designed to measure the general level of activity and exploratory behavior, mainly under stressful conditions (nonhome cage environment), was performed using the ActiMot2 system (TSE systems). The mice were placed in conventional cages without bedding, water, or food. Activity was measured for 15 min. The same parameters were read as in the home cage activity test, and the same software was used. Anxiety levels were assessed by counting the number of beam breaks in the peripheral parts of the cage, that is, the number of fine and ambulatory movements in these areas. The test was performed at 12 and 19 months of age.

### 2.12 Immunohistochemistry

At age 12 months, HD/100CAG mice, HD/100Q mice, and their wild‐type littermates were subjected to cardiac perfusion with PBS to remove all blood and were then perfused with 4% paraformaldehyde solution. The fixed mouse brains were isolated, and after 24 h, they were transferred to 30% sucrose for 72 h. Then, 8‐μm tissue sections were prepared using a cryostat at −21°C and mounted on SuperFrost Plus slides (Thermo Scientific). The brain sections were processed for RAN protein detection using rabbit anti‐human HD‐polySer‐Ct and HD‐polyAla‐Ct polyclonal antibodies (both from Sigma–Aldrich) as described previously with some modifications.^31^, ^33^ Briefly, the sections were fixed in 4% paraformaldehyde in PBS for 15 min at RT, rinsed in PBS, and subjected to a triple antigen retrieval process, which involved (1) proteinase K treatment (1 μg/mL) proteinase K in 1 mM CaCl_2_ and 50 mM Tris buffer (pH = 7.6) for 40 min at 37°C; (2) incubation in 10 mM EDTA (pH = 6.5) for 15 min in a water bath heated to 95°C; and (3) formic acid treatment for 7 min at RT. The slides were then washed with distilled water for 10 min and incubated in 3% H_2_O_2_ in methanol for 10 min to block endogenous peroxidase activity. Nonspecific Ig binding was blocked by incubation with 10% horse serum (Sigma) in PBS for 60 min, followed by incubation with anti‐HD‐polyAla‐Ct and anti‐HD‐polySer‐Ct polyclonal antibodies (1:20 000 both) in 10% horse serum in PBS overnight at 4°C. The slides were washed in PBS three times and incubated with ImmPRESS‐HRP Horse Anti‐Rabbit IgG Polymer Reagent (Vector Laboratories) for 30 min at RT. Immunoreactivity was visualized with ImmPACT DAB EqV reagents (Vector Laboratories) for 1 min. The slides were washed in water for 10 min and later counterstained with hematoxylin (Sigma) for 5 min. After the last wash in water, the slides were dehydrated, and coverslipped using CV Mount (Leica). Images were captured with a Leica DMI4000 inverted microscope.

### 2.13 NanoString nCounter analysis and data processing

Total RNA was extracted from frozen mouse striatal tissues with an RNeasy Lipid Tissue Mini Kit (Qiagen). The tissues were homogenized in Qiazol Lysis Reagent until a homogeneous lysate was obtained, as described above. The genomic DNA was digested on a column with an RNaseFree DNase Set (Qiagen). The RNA concentration and quality were determined using a spectrophotometer (DeNovix) and a fluorometer (Qubit RNA BR Assay Kit, Thermo Fisher Scientific), and RNA integrity was evaluated via 1% agarose gel electrophoresis and staining with ethidium bromide.

Transcriptomic analysis was performed on the nCounter system using a Mouse Neuropathology Panel (measuring the expression of 760 neuropathology‐related mouse genes and 10 reference genes, nCounter Analysis System FLEX, NanoString Technologies) according to the manufacturer's instructions (nCounter XT Assay User Manual, July 2016, MAN‐10023‐11). For the NanoString nCounter XT CodeSet Gene Expression Assay, we used 100 ng of total RNA (per sample) purified from 21‐month‐old WT, HD/100Q, and HD/100 CAG mice (4 animals per group).

Following data collection, the raw data were processed using nSolver 4.0 software (NanoString Technologies) for background correction, normalization, and ratio estimation. The background correction was estimated by a defined threshold count value of 25. The data were normalized to the geometric mean of the average count of six positive controls included in the assay and 10 housekeeping genes included in the neuropathology panel. The following comparisons between the 21‐month‐old animals were performed to estimate the fold change (FC) in expression: HD/100Q mice vs. WT mice and HD/100CAG mice vs. WT mice. Genes with FC > 1 and FC < 1, as well as *p* values ≤.05, were considered significantly up and downregulated, respectively. For genes for which the FC was less than one, the (negative) reciprocal value is shown in Table (e.g., FC for *Car2* (HD/100Q mice) was 0.577, and a decrease of 42.3% compared with WT mice is reported as a −1.733‐fold change).

Ingenuity pathway analysis (IPA) (Qiagen Inc., https://digitalinsights.qiagen.com/IPA) was used to identify canonical pathways associated with the identified differentially expressed genes (DEGs). The gene ID, *p* value, and fold change in the DEGs identified by nSolver analysis were inserted into IPA core analysis function,^40^ and a −log (*p* value) >1.3 was used as the threshold.

PPI networks were visualized using the STRING version 12 database (https://string‐db.org/, Szklarczyk et al.^41^), and the cytoHubba plugin of Cytoscape software version 3.10.1 (https://cytoscape.org/) was applied to identify the top 10 hub genes according to the maximal clique centrality (MCC) method. The representation factor (RF) and corresponding *p* value were used to quantify the significance of overlap between dysregulated genes in HD/100CAG and HD/100Q mice and were calculated using the tool found at http://nemates.org/MA/progs/overlap_stats.html. RF is the number of overlapping genes divided by the expected number of overlapping genes drawn from two independent groups. An RF > 1 indicates more overlap than expected by chance for two independent groups of genes or events, and an RF < 1 indicates less overlap than expected. The probability of finding four overlapping genes was determined using the hypergeometric probability formula.

### 2.14 Statistical analysis

All the statistical analyses were performed using GraphPad Prism 8 software, and the statistical tests used for the molecular and behavioral data are indicated in each figure legend. Data are expressed as the mean ± SEM. The number of animals/samples included in each experiment is given in Figure 1. Only statistically significant results are marked in the figures with *p < .05; **p < .01; ***p < .001; ****p < .0001 (the same ranges also apply for ^^^ and ^#^), and close to statistically significant results (.05 < *p* < .1) have *p* values given.

## 3 RESULTS

### 3.1 Generation of HD mouse lines expressing translated or nontranslated mutant *HTT*

**FIGURE 2:**
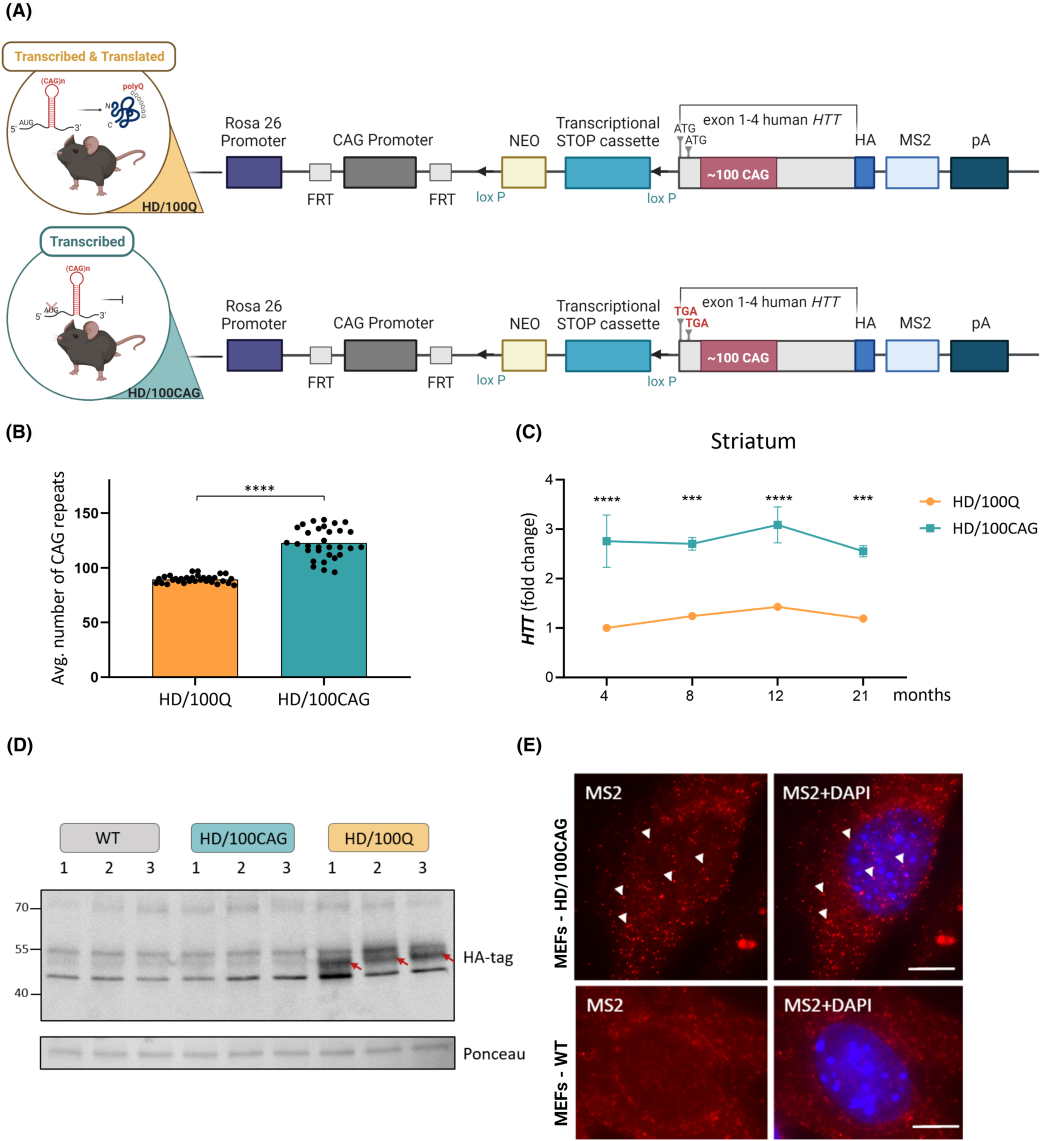
Characterization of the *HTT* transgene in the generated mouse models. (A) Expression cassettes introduced into the *Rosa*26 locus in the parental lines of the HD/100Q and HD/100CAG knock‐in mice, with the main elements indicated. The only difference between these expression cassettes was the presence of two TGA codons, marked in red for the HD/100CAG cassette, instead of naturally occurring ATG codons. The Cre‐loxP system enabled the removal of the transcriptional STOP cassettes by crossing with Cre‐expressing mice. For more details, see the text. (B) Characteristics of the mutant *HTT* transgenes in homozygous HD/100Q and HD/100CAG mice. The average number of CAG repeats in the two alleles of the transgene in each mouse from the analyzed cohort is plotted (*n* = 31 HD/100CAG, *n* = 31 for HD/100Q). The average length of CAG tracts between models was compared using an unpaired t test; ****p < .0001. (C) Quantification of *HTT* transgene expression in the striatum of HD/100Q and HD/100CAG mice at the indicated ages by RT–qPCR. *Atp5b* and *Gapdh* were used as reference genes. Mean ± SEM; Two‐way ANOVA with Bonferroni's post hoc test; ***p < .001; ****p < .0001. (D) Representative blot of immunoprecipitated samples isolated from 21‐month‐old mice, HD/100Q model showing the presence of the transgenic HTT. Immunoprecipitation of whole cortical lysates was performed using anti‐HA‐tag agarose, and the membranes were probed with an anti‐HA‐tag antibody. Bands corresponding to mutant HTT fragments are indicated by red arrows. The mutant *HTT* alleles in individual HD/100Q mice (1, 2, and 3) included 78/89, 88/101, and 88/93 CAG repeats, respectively. A strip of the Ponceau S‐stained membrane is shown for verification of consistency in sample preparation. (E) Representative images of smFISH of mutant *HTT* RNA in MEFs isolated from E16.5 HD/100CAG mouse embryos and negative control MEFs isolated from WT mice. Exemplary specific spots of *HTT* RNA are indicated by the white triangles. Additional results are presented in Figure S4. Nuclei were stained with DAPI. Scale bar = 10 μm.

To develop models suitable for investigating the contribution of RNA toxicity in HD, we designed two mouse lines expressing an N‐terminal fragment of human *mHTT* cDNA spanning exons 1–4 (Text S1) under the control of the CAG synthetic promoter. The cDNA fragments in the two lines differed in the presence of two ATG start codons in exon 1, resulting in either the exclusive production of *HTT* RNA (HD/100CAG model) or the canonical expression of the transgene, that is, the production of *HTT* RNA accompanied by the production of a 257‐amino acid HTT fragment (HD/100Q model) (Figure 2A). Furthermore, an HA tag and MS2 aptamer were placed at the 3′ end of the HTT fragment for mHTT protein detection and transgene RNA visualization, respectively. To ensure stable and ubiquitous expression, the transgene sequence was cloned and inserted into a *Rosa26* locus targeting vector. The potential promoter switch between the CAG promoter and the native *Rosa26* promoter was enabled by FRT sites flanking the H19 insulator sequence and the CAG promoter (Figure 2A). In addition, a loxP‐flanked neo/STOP cassette was inserted downstream of the *Rosa26* and CAG promoters to prevent potential toxic effects of transgene expression during the initial steps of generating these HD mouse models. The developed *Rosa26* targeting vector (see Text S2 and Figure S1 for more details) was injected into blastocysts, and the resulting chimeric mice were backcrossed with C57BL/6N mice for three generations and subsequently mated with Cre transgenic mice to excise the transcriptional neo/STOP cassette.

A cohort of male homozygous animals was generated, and a total of 31 HD/100CAG and 31 HD/100Q mice were analyzed. Wild‐type (WT) littermates were used as controls. The timeline of the molecular and behavioral analyses included several main timepoints (Figure 1).

### 3.2 *HTT* transgene characterization

First, we analyzed the number of CAG repeats in the *HTT* transgenes in all the mice in the cohort. Specifically, the *HTT* alleles in HD/100Q mice had an average of 90 CAG repeats (ranging from 84 to 97), whereas those in HD/100CAG mice had an average of 123 CAG repeats (ranging from 96 to 144) (Figure 2B). Generally, the repeat tracts of the transgenes in the HD/100CAG mice were both longer and more variable than those in the HD/100Q mice but to the extent of allowing direct comparison of the models, as previously investigated in other models.^42^, ^43^, ^44^

The transcript level of the *HTT* transgene in the striatum was relatively stable in HD/100Q and HD/100CAG mice based on data obtained at four timepoints (Figure 2C). We consistently observed an approximately threefold higher transcript level in HD/100CAG mice than in HD/100Q mice. Similar results were obtained for the cortex and hippocampus (Figure S2A). Moreover, in the brain tissue of HD/100Q mice, the transcript levels of the transgene and the endogenous mouse *Htt* were similar (based on similar delta Ct values determined by RT–qPCR; Figure S2B), indicating relatively low expression of the transgene. Generally, the change in endogenous *Htt* expression with age was not substantially different between HD/100Q or HD/100CAG mice and WT animals (Figure S2C).

Given the low expression of the transgene and the known problems with visualization of the N‐terminal mutant HTT fragments by western blotting^43^, ^45^, ^46^ we decided to detect mHTT proteins in the HD/100Q model using immunoprecipitation (IP). We detected the expected mHTT fragments after IP of cortical lysates with anti‐HA‐tag agarose (Figures 2D and S3). The migration of detected proteins from individual mice corresponded to the number of Q repeats in the protein (see Figure 2D legend for details). As expected, transgenic HTT was not detected in the immunoprecipitates of cortical tissues from HD/100CAG or WT mice (Figures 2D and S3).

For visualization of MS2‐tagged mutant *HTT* transcripts in cells, we performed single‐molecule fluorescent in situ hybridization (smFISH) in MEFs isolated from HD/100CAG mice (Figure 2E). As a negative control, WT MEFs were used. A DNA probe against the MS2 sequence with 11 binding sites for one *HTT* transgene transcript was used. Numerous bright cytoplasmic and nuclear spots, each corresponding rather to a single mRNA, were observed in MEFs from HD/100CAG mice but were not detected in MEFs from WT mice (Figures 2E and S4). The tagged *HTT* transcripts were predominantly cytoplasmic; however, several spots were detected in the nucleus. Nevertheless, no clear *HTT* RNA foci/clusters were observed in the analyzed cells.

### 3.3 Changes in the organ weight of HD mice

There was no substantial difference in body mass between HD/100CAG or HD/100Q mice and WT mice at 4 to 21 months of age (Figure S5). Compared to their WT counterparts, HD model mice neither lost nor gained weight over time. At four timepoints, the mice were sacrificed, and their organs (brains, hearts, kidneys, and spleens) were weighed. There were no substantial differences in brain weight; only at 8 months of age were significant differences reported between the HD/100Q and HD/100CAG mice (Figure S5B). The hearts of the HD/100CAG mice were generally heavier than those of the WT mice, especially at 12 months of age (Figure S5C). No significant differences were reported in kidney weight (Figure S5D), whereas the differences in spleen weight between HD/100CAG and WT mice and between HD/100CAG and HD/100Q mice were significant throughout the experiment. The spleens of HD/100CAG mice were on average lighter than those of WT and HD/100Q mice. A significant difference was observed at the endpoint of the experiment: the spleen weight of HD/100Q mice (mean 0.12 g) was substantially greater than that of WT (mean 0.07 g) and HD/100CAG (mean 0.07 g) mice (Figure S5E), suggesting ongoing chronic inflammation in HD/100Q mice resulting in splenomegaly.

We also assessed the serum concentrations of alkaline phosphatase, cholesterol, uric acid, and alanine aminotransferase (Figure S6), alongside the urinal level of homocysteine data not shown in 12‐month‐old mice. No statistically significant differences were found (Figure S6), suggesting a lack of substantial metabolic alterations in the investigated mice.

### 3.4 Locomotor deficits in HD mice

**FIGURE 3:**
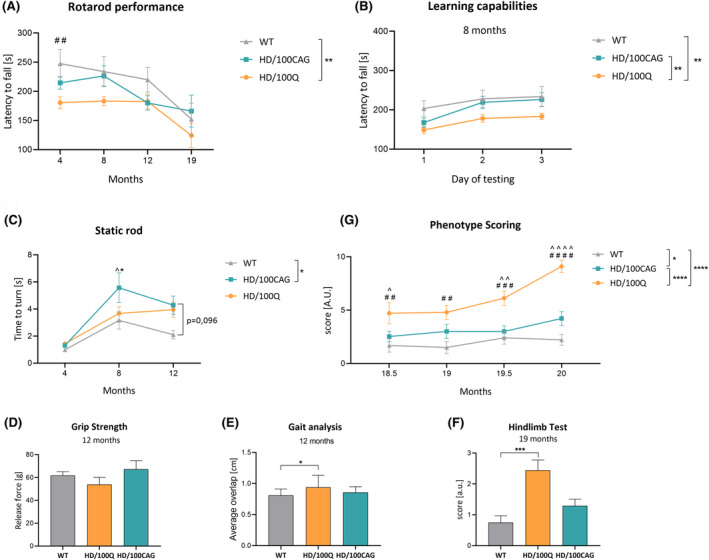
Locomotor activity of HD/100CAG and HD/100Q mice. (A) The rotarod test was used to evaluate the motor coordination and balance of mice at 4, 8, 12, and 19 months of age, and the latency to fall is presented. (B) The rotarod test was also used to assess the cognitive capabilities (learning) of the mice, which was measured by comparing performance on the test on three separate days; practice trials were performed on the first 2 days, while the test data presented in (A) were collected on the third day (the test day). (C) Results of the static rod test (time to turn around) at 4, 8, and 12 months of age. (D) Grip strength at 12 months of age. (E) Results of gait analysis at 12 months of age. (F) Assessment of motor deficits by the hindlimb test at 19 months of age. (G) Phenotyping scores at 18.5–20 months of age. The number of animals per group is shown in Figure 1. The statistical results presented in the figure legend were obtained by two‐way ANOVA (main effect of genotype) followed by Tukey's test; while ^#^, *, ^^^ indicate a simple effect within rows according to Tukey's post hoc test (^#^HD/100Q mice vs. WT mice; *HD/100CAG mice vs. WT mice; ^^^HD/100Q mice vs. HD/100CAG mice). The data in panels D–F were analyzed using one‐way ANOVA with Holm–Sidak's multiple comparisons test. *p < .05; **p < .01; ***p < .001; ****p < .0001 (the same ranges also apply for ^^^ and ^#^).

Impaired motor performance is one of the main symptoms of HD and is also recapitulated by many mouse models of HD.^47^ To quantify motor deficits in HD/100CAG and HD/100Q mice, we performed a rotarod test at 4, 8, 12, and 19 months of age. The motor performance of the HD/100Q mice was significantly affected at the first timepoint (4 months), as the latency to fall on the rotarod was reduced by almost 75 s for HD/100Q mice compared to WT mice (Figure 3A). There was a tendency toward impaired motor performance in HD/100Q mice at all analyzed timepoints, and the effect of genotype was statistically significant (presented in the figure legends). To assess the learning abilities of the model mice, the rotarod test was conducted consecutively for 3 days. The results showed that at 8 months of age, there were no discernible differences in learning abilities among the mice from tested groups (as indicated by the fact that the curves have similar slopes), but the motor performance of the HD/100Q mice differed significantly from that of the other groups. The rotarod test revealed no significant differences in performance between the HD/100CAG mice and the WT mice (Figure 3A,B).

To further assess motor function, we performed a static rod test. Already at 8 months of age, the HD/100CAG mice exhibited a significantly prolonged turning time compared to the other groups (Figure 3C). At 12 months of age, both the HD/100Q and HD/100CAG mice displayed markedly inferior performance compared to the WT mice, evidenced by their prolonged time needed to turn on the static rod (Figure 3C). While the WT animals executed turns in approximately 2 s, mice from the two HD models required twice the duration to complete the turn; however, this difference did not attain statistical significance.

Moreover, to assess other aspects of the locomotor function, we performed grip strength, gait analyses, and the hindlimb test (Figure 3D–F). Gait analysis and the hindlimb test, performed at 12 and 19 months of age, respectively, revealed statistically significant differences between the HD/100Q mice and WT mice (Figure 3E,F). Furthermore, a phenotype scoring system was used to assess disease severity in the generated HD models at the end of the study (for 6 weeks, 4 timepoints); specifically, the degree of kyphosis and performance on the ledge, gait, and hindlimb tests were scored (Figure 3G). Our observations of visible motor impairment in the HD/100Q model were confirmed by the scoring results; HD/100Q mice exhibited significant phenotypic alterations, including a visibly curved back, an imbalanced gait, and poor coordination. At each of the analyzed timepoints, HD/100Q mice exhibited deficits, with disease phenotypes worsening over the course of the study (an increase from an average of ~5 points to ~9 points over 6 weeks; 12 points was the maximum score that could be awarded; the score of the WT mice remained stable over time at ~2 points) (Figure 3G). Also, HD/100CAG mice exhibited worse scores compared to WT mice (an increase in score from 2.5 points to 4.2 points over 6 weeks) in all tests at a similar level. The statistical analysis of the phenotype scores indicated a genotype effect, with the phenotype scores of HD/100CAG mice significantly differing from those of WT and HD/100Q mice (Figure 3G).

### 3.5 Increased activity and anxiety in HD mice

**FIGURE 4:**
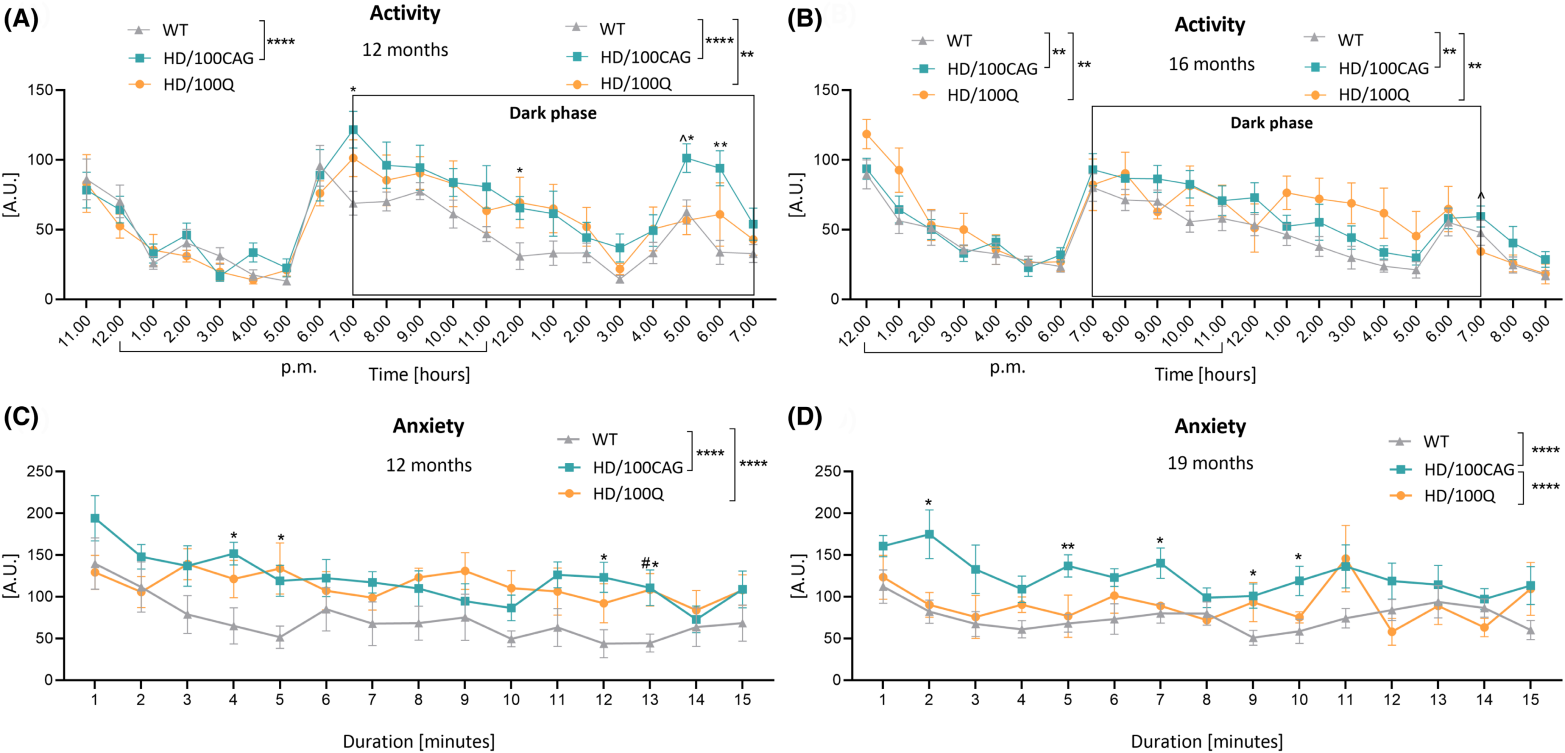
Activity of HD/100CAG and HD/100Q mice in the home cage and open field tests. (A, B) Home cage activity was assessed ~20 h with the ActiMot system at 12 (A) and 16 (B) months of age for. The dark phase is indicated, and a separate statistical analysis was performed for these data. (C, D) Anxiety behaviors were evaluated for 15 min in nonhome cages (no feed, water, or bedding) with the ActiMot system at 12 (C) and 19 (D) months of age (^#^HD/100Q mice vs. WT mice; *HD/100CAG mice vs. WT mice; ^^^HD/100Q mice vs. HD/100CAG mice). The number of animals per group is shown in Figure 1. The statistical results presented in the figure legend were obtained by two‐way ANOVA (main effect of genotype) followed by Tukey's test; while ^#^, *, ^^^ indicate a simple effect within rows according to Tukey's post hoc test. *p < .05; **p < .01; ***p < .001; ****p < .0001 (the same ranges also apply for ^^^ and ^#^).

As psychiatric symptoms such as depression and anxiety are reported in HD patients,^48^, ^49^ we utilized a precise cage monitoring system to assess mouse movement. The activity of 12‐month‐old (Figure 4A) and 16‐month‐old (Figure 4B) mice was analyzed using the ActiMot system. Analysis of the data collected over ~20 h revealed statistically significant differences in activity between the HD/100CAG and WT mice (Figure 4A,B). When we specifically looked at the data gathered during the dark phase, we observed significant differences in activity at both timepoints in HD/100CAG and HD/100Q mice (Figure 4A,B). Both models were more active than WT mice in the dark phase at the 12‐ and 16‐month timepoints, and this difference was particularly significant in HD/100CAG mice at 12 months.

We also used the ActiMot system and non‐home cages to perform open‐field tests on 12‐month‐old (Figure 4C) and 19‐month‐old (Figure 4D) mice. The purpose of these tests was to assess the behavioral response of animals under stress conditions. Behavior was analyzed over 15 min, during which the mice did not have access to bedding, feed, or water, to assess novel environment recognition. Both HD/100CAG and HD/100Q mice exhibited higher anxiety levels than WT mice at 12 months of age (Figure 4C). Moreover, 19‐month‐old HD/100CAG mice exhibited higher anxiety levels than the other groups, what was also shown by significant differences between the HD/100CAG and WT mice at several timepoints (min) (Figure 4D). Multiple comparisons of genotypes revealed differences not only between HD/100CAG and WT mice but also between HD/100CAG and HD/100Q mice.

### 3.6 RAN‐translated products are not detected in HD mice

One of the postulated mechanisms by which mutant RNA can trigger the pathogenesis of HD is the induction of RAN translation.^24^, ^50^, ^51^ The occurrence of this unconventional form of translation has been analyzed in three different mouse models of HD, and the presence of RAN‐translated proteins was successfully detected in two of them.^31^, ^33^, ^52^ In addition, RAN‐translated proteins were detected in the *postmortem* brain tissues of HD patients.^33^ Thus, we decided to determine whether RAN translation occurs in the HD models generated in this study. We expected that this process could be enhanced in the HD/100CAG model, in which the canonical translation of the *HTT* transcript does not occur. The materials used for the analysis were brain tissue samples from 12‐month‐old mice and whole cortical, striatal, and hippocampal lysates from 21‐month‐old mice. Using available antibodies recognizing HD‐polyAla‐Ct and HD‐polySer‐Ct RAN‐translated proteins, we were unable to find any specific signals in either brain sections (Figure S7) or brain lysates from HD/100Q and HD/100CAG mice (data not shown). Additionally, in immunoprecipitates from HD/100CAG mice, we did not detect any specific signals for RAN‐translated polyQ proteins (due to the HA tag) (Figures 2D and S3). We did not rule out that RAN translation occurs in our models but at an undetectable level. This may be related to low levels of mutant *HTT* transcripts in the investigated models. Another possible scenario explaining the observed results may be that aberrantly spliced *HTT* transcripts are more prone to RAN translation in HD, and in our models, aberrantly spliced transcripts were not present due to the transgene used.

### 3.7 Neuropathology‐associated dysregulated genes in HD/100CAG mice partially overlap with those in HD/100Q mice

HD and other neuropathological disorders are strongly associated^22^ with disturbances in gene expression in particular brain regions and cell types.^53^ We investigated the neuropathology‐related gene expression patterns in the striatum with a nCounter analysis system. For this analysis, we compared total RNA isolated from the tissues of 21‐month‐old homozygous male mice that had developed an HD phenotype with that isolated from the tissues of their WT littermates.

**FIGURE 5:**
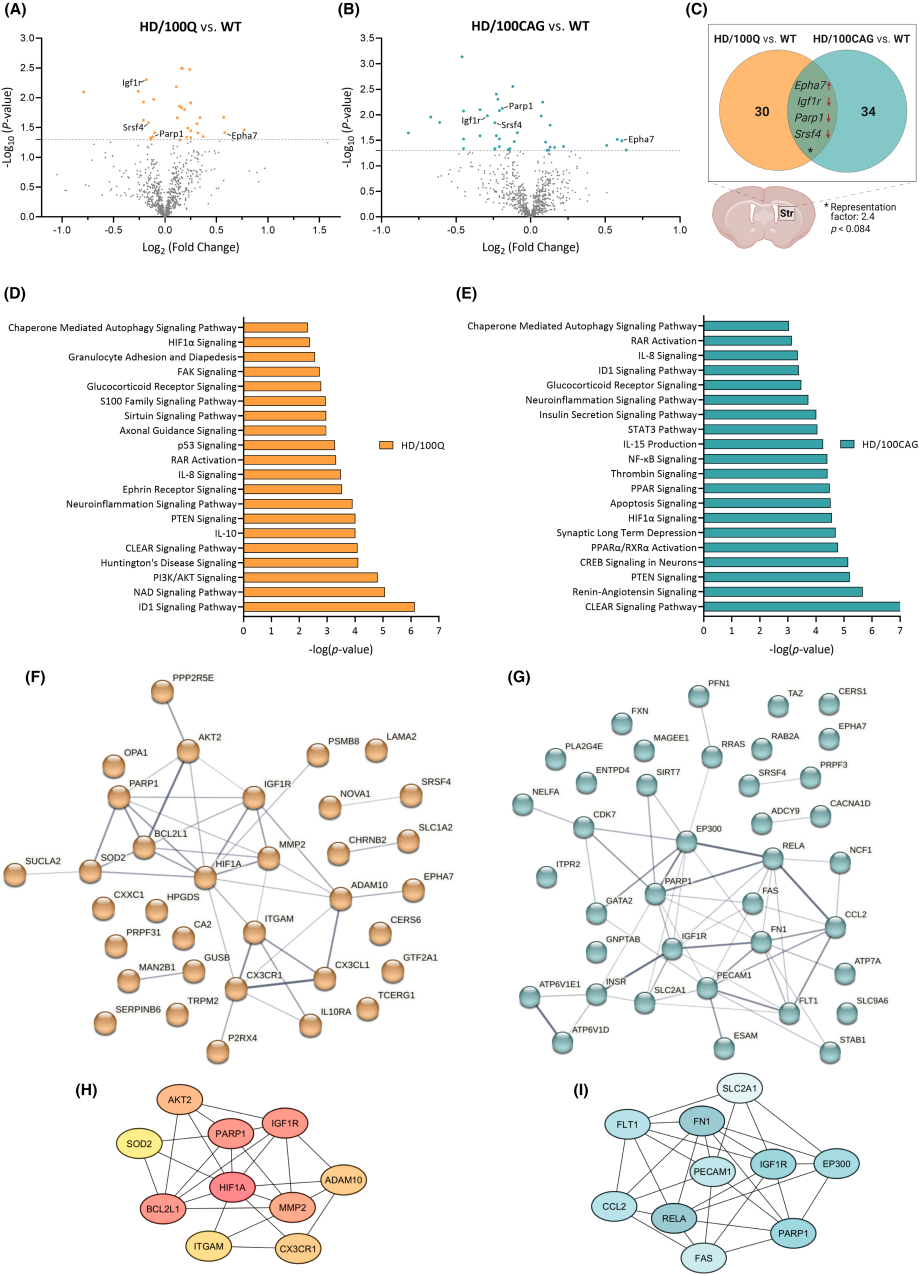
Differential expression of genes in the HD/100Q and HD/100CAG models. Gene expression in samples isolated from the striatum of 21‐month‐old mice was analyzed with a NanoString nCounter Mouse Neuropathology Panel. (A, B) Volcano plots showing differential gene expression between HD/100Q mice and WT mice (A) and between HD/100CAG mice and WT mice (B). *n* = 4. DEGs with a *p* value ≤.05 are indicated in orange or blue. (C) Venn diagram indicating the number of transcripts whose levels were significantly changed in one or both models. Genes that showed similar changes in expression in both models included *Parp1*, *Igf1r*, and *Srsf4*, which were downregulated, and *Epha7*, which was upregulated. (D, E) Canonical pathways identified by IPA with the core analysis function. Histograms displaying the top 20 significantly altered canonical pathways in HD/100Q mice compared with WT mice (D) and in HD/100CAG mice compared with WT mice (E). (F, G) Network analysis of proteins encoded by the dysregulated genes (indicated by the colored dots in A and B) in HD/100Q mice compared with WT mice and (G) in HD/100CAG mice compared with WT mice (F) using the STRING v. 12 database for humans. The edges represent protein–protein associations, and the line thickness indicates the confidence score. (H, I) Networks of the top 10 hub genes according to the maximal clique centrality (MCC) method for the HD/100Q (H) and HD/100CAG (I) models. The darker the color is, the stronger the association with the other genes. In the PPI network, the human orthologs of Car2, Serpinb6a, and Ccl12 are assigned slightly different names: CA2, SERPINB6, and CCL2, respectively.

A total of 34 and 38 genes were found to be differentially expressed in HD/100Q mice compared with WT mice and in HD/100CAG mice compared with WT mice, respectively (Table 1, Figure 5A,B). Specifically, 23 genes were upregulated, and 11 were downregulated in the striatum in the HD/100Q mice, whereas in the HD/100CAG mice, we observed the upregulation of 13 genes and the downregulation of 25 genes. Moreover, we identified four genes that were dysregulated and showed the same direction of change in expression in both models (Figure 5C). The genes that showed similar changes in expression in both models included poly(ADP‐ribose) polymerase 1 (*Parp1*), insulin growth factor 1 receptor (*Igf1r*), and serine and arginine‐rich splicing factor 4 (*Srsf4*), which were downregulated, and EPH receptor A7 (*Epha7*), which was upregulated. The RF between the two models for this set of overlapping genes was 2.4 (*p* < .084), which suggests greater overlap than expected by random chance (Figure 5C).

**TABLE 1:** Genes that were significantly differentially expressed between HD mice and control mice (*p* ≤ .05). For genes for which the FC was less than one, the (negative) reciprocal value is shown (e.g., FC for Car2 (HD/100Q mice) was 0.577, and a decrease of 42.3% compared with WT mice is reported as a −1.733‐fold change).

mRNA	Fold change HD/100Q vs. WT	*p* Value	mRNA	Fold change HD/100CAG vs. WT	*p* Value
*Psmb8*	1.701	0.034	*Entpd4*	1.558	0.049
*Epha7*	1.491	0.038	*Epha7*	1.529	0.032
*Mmp2*	1.48	0.021	*Ccl12*	1.495	0.030
*P2rx4*	1.293	0.045	*Pla2g4e*	1.425	0.040
*Lama2*	1.264	0.021	*Cers1*	1.164	0.042
*Gusb*	1.246	0.039	*Atp6v1e1*	1.118	0.043
*Cx3cr1*	1.246	0.039	*Slc9a6*	1.095	0.016
*Il10ra*	1.237	0.027	*Cdk7*	1.091	0.043
*Gtf2a1*	1.193	0.012	*Fxn*	1.088	0.049
*Hpgds*	1.193	0.012	*Magee1*	1.076	0.049
*Itgam*	1.19	0.036	*Atp6v1d*	1.073	0.034
*Serpinb6a*	1.188	0.047	*Pfn1*	1.056	0.006
*Prpf31*	1.185	0.003	*Rab2a*	1.049	0.011
*Nova1*	1.163	0.032	*Gnptab*	−1.064	0.022
*Man2b1*	1.154	0.046	*Ep300*	−1.078	0.033
*Chrnb2*	1.141	0.016	*Nelfa*	−1.083	0.003
*Trpm2*	1.127	0.003	*Prpf3*	−1.1	0.046
*Hif1a*	1.121	0.003	*Taz*	−1.108	0.048
*Tcerg1*	1.114	0.015	*Parp1*	−1.145	0.008
*Sucla2*	1.107	0.050	*Rras*	−1.157	0.008
*Ppp2r5e*	1.105	0.014	*Sirt7*	−1.16	0.030
*Opa1*	1.088	0.022	*Atp7a*	−1.161	0.042
*Sod2*	1.08	0.007	*Ncf1*	−1.167	0.005
*Cxxc1*	−1.071	0.039	*Insr*	−1.17	0.004
*Adam10*	−1.08	0.011	*Srsf4*	−1.177	0.026
*Parp1*	−1.085	0.045	*Adcy9*	−1.178	0.014
*Bcl2l1*	−1.104	0.047	*Cacna1d*	−1.181	0.046
*Akt2*	−1.105	0.050	*Rela*	−1.182	0.047
*Srsf4*	−1.12	0.026	*Igf1r*	−1.221	0.010
*Igf1r*	−1.13	0.005	*Itpr2*	−1.267	0.008
*Cers6*	−1.158	0.012	*Stab1*	−1.27	0.026
*Cx3cl1*	−1.158	0.024	*Esam*	−1.362	0.008
*Slc1a2*	−1.198	0.008	*Gata2*	−1.362	0.030
*Car2*	−1.733	0.008	*Pecam1*	−1.367	0.046
			*Flt1*	−1.372	0.001
			*Slc2a1*	−1.522	0.014
			*Fn1*	−1.589	0.011
			*Fas*	−1.764	0.023

According to IPA of the DEGs with the core analysis function, 118 canonical pathways were altered in the HD/100Q mice, and 198 were altered in the HD/100CAG group (*p* < .05). A total of the same 69 canonical pathways were found to be altered in both models compared to controls. Among these pathways, many were associated with the inflammatory response. Other identified signaling pathways were related to apoptosis, autophagy, DNA repair, cell energy metabolism, nervous system development, and synaptic function. The top 20 pathways among all dysregulated canonical pathways associated with neurodegeneration in both models are shown in Figure 5D,E (for a complete list of altered canonical pathways in both models and additional information, see Supplementary File S1 and Text S3).

We analyzed the PPI network of the human orthologs of the identified dysregulated genes and indicated the top 10 hub genes (Figure 5F–I). Of the 34 identified transcripts whose expression was dysregulated in the HD/100Q model, 23 exhibited protein‐level interactions (interaction score: confidence, 0.400; PPI enrichment *p* value, 5.65e−05; Figure 5F). Functional enrichment analysis of the PPI network revealed a wide range of biological processes associated with nervous system function, among other processes. In particular, STRING analysis highlighted the significant involvement of the PPI network in several biological processes that are known to be disrupted in HD, including apoptotic processes associated with the inflammatory response such as “regulation of hippocampal neuron apoptotic process,” “positive regulation of microglial cell migration,” “positive regulation of I‐kappaB phosphorylation,” and “macrophage migration.” These processes as well as “synapse pruning” and “autocrine signaling” are regulated mainly by CX3CL1, CX3CR1, and ITGAM in this network. Other notable biological pathways included the response to oxidative stress‐related processes such as “oxygen homeostasis,” “regulation of oxidative stress‐induced intrinsic apoptotic signaling pathway,” and overall “regulation of oxidative stress‐induced cell death,” especially in neurons, which mainly involves PARP1, HIF1A, and SOD2. All proteins mentioned above, along with EPHA7, BCL2L1, and AKT2, ultimately regulate neuronal apoptosis and cell death. Processes such as “positive regulation of hormone biosynthetic process,” “axonal transport of mitochondrion,” and “response to amyloid‐beta” also play crucial roles in normal brain function. Dysfunction of these biological processes can contribute to various neurological disorders. In the HD/100Q model, these alterations are expected to result from the combined pathogenic effects of the mutant protein and transcript.

Analysis of striatal samples from HD/100CAG mice with nCounter revealed 38 DEGs (compared with WT mice), 27 of which exhibited protein‐level interactions (interaction score: confidence, 0.400; PPI enrichment *p* value, 7.08 e−06; Figure 5G). According to the STRING database, these DEGs are predicted to play a role in several biological processes, as they were significantly enriched in “negative regulation of vascular endothelial cell proliferation” (*FLT1* and *CCL2*) and “positive regulation of PI3K signaling” (*INSR, IGF1R, FN1, FLT1, NCF1*), which coordinates a variety of complex events, including changes in neuronal development, growth, survival, and energy metabolism, and orchestrates immune defense mechanisms.^54^ Other less significantly enriched biological processes identified by STRING analysis included “regulation of ATP metabolic process,” “cellular response to reactive oxygen species,” and “cellular response to chemical and oxidative stress.” It can therefore be assumed that the dysregulation of the above biological processes in the HD/100CAG model was caused by the toxic effect of the mutant transcript.

The hub genes identified from the PPI network using the MCC algorithm of the CytoHubba plugin in Cytoscape are shown in Figure 5H,I. According to the MCC ranking, the top 10 highest‐scored genes in the HD/100Q model (*IGF1R, BCL2L1, AKT2, PARP1, ADAM10, SOD2, HIF1A, ITGAM, CX3CR1, MMP2*; Figure 5H) and HD/100CAG model (*FN1, RELA, IGF1R, PARP1, EP300, FLT1, PECAM1, CCL2, FAS, SLC2A1*; Figure 5I) mostly overlapped with the most significantly enriched biological processes identified from the STRING database reported above. Two hub genes, *PARP1* and *IGF1R*, were identified in both models.

## 4 DISCUSSION

A group of several dozen rare diseases, mainly affecting the nervous system, are caused by the expansion of repeated tracts of specific sequence motifs, mostly trinucleotides.^50^, ^55^ Since the discovery of the causative mutations of these diseases, diverse molecular mechanisms driving disease pathogenesis have been described.^56^ Concerning mutant gene expression, these mechanisms include dominant gain‐of‐function or loss‐of‐function effects, which occur at the RNA or protein level and depend on the location of the mutation within the disease‐associated gene (untranslated or translated regions). Expanded CAG repeats are mostly related to diseases due to their presence in the translated regions of specific genes. Therefore, it is well established that mutant protein gain‐of‐function mechanisms are involved in polyQ disease pathogenesis. Nevertheless, repeat tracts are also known to cause disease when present in untranslated regions; therefore, RNA gain‐of‐function mechanisms have been suggested for mutant genes with translated CAG tracts. The concept of RNA toxicity in polyQ diseases has been studied for more than 15 years, and numerous studies have shown that mutant CAG repeat‐containing mRNAs cause cellular effects that drive neurodegeneration in combination with toxic polyQ tract‐containing proteins.^23^, ^24^, ^57^ RNA toxicity in polyQ diseases has already been investigated to some extent in mice utilizing transgenes with untranslated expanded CAG repeats. Hsu et al.^58^ analyzed the effects of the expression of untranslated mutant CAG tracts (located in the 3′UTR of EGFP) in muscles and testes (the expression was not driven in the brain). The authors observed significant alterations in the muscles of animals overexpressing a transgene containing 200 CAG repeats compared to those expressing a transgene with 23 CAG repeats. In another study, Yang et al.^52^ introduced an *HTT* transgene with a deletion before the CAG repeat tract into mice to prevent canonical translation. The animals showed no alterations in motor functions up to 9 months of age and no significant transcriptional dysregulation at 6 months of age.

**FIGURE 6:**
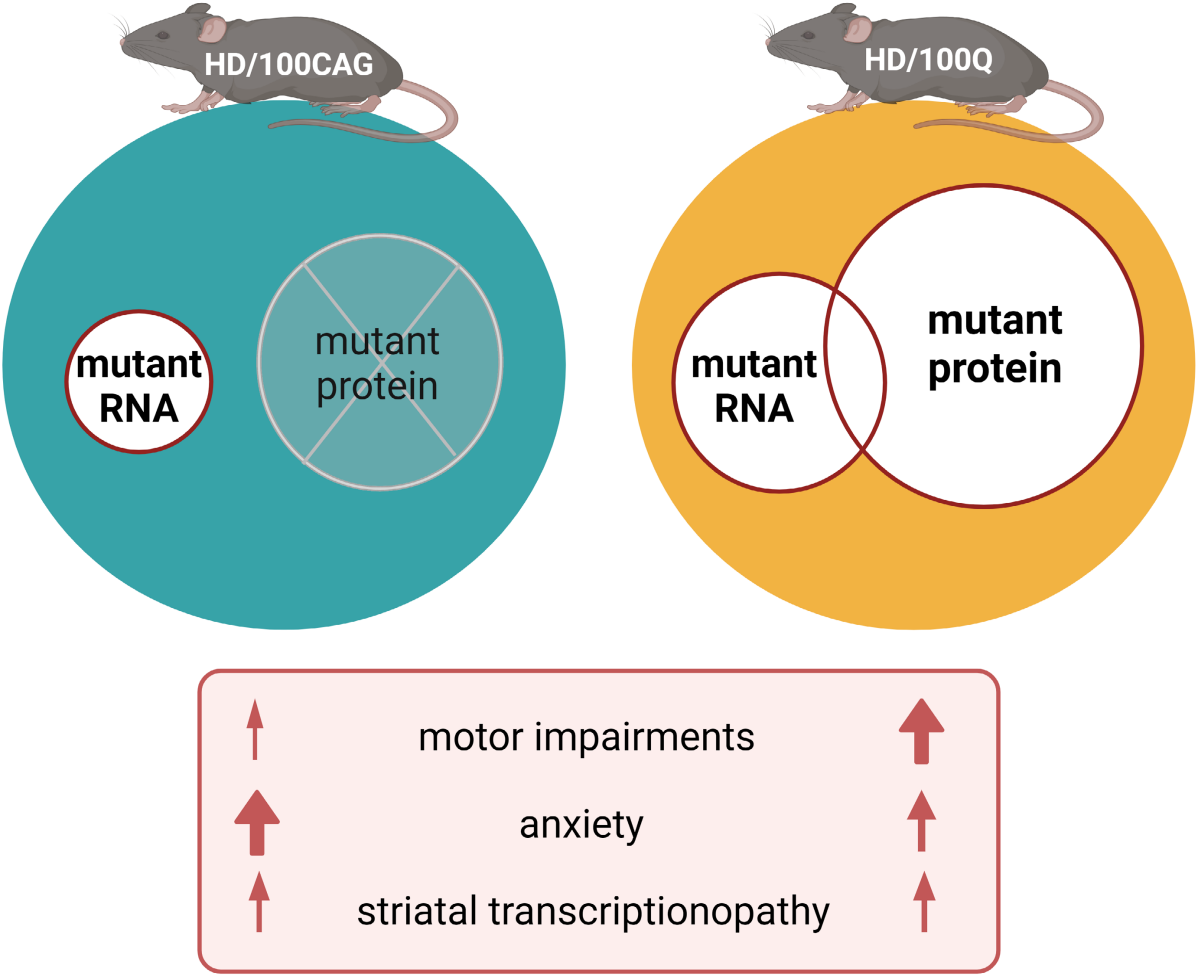
Our novel HD mouse models enable the study of alterations in pathways caused by mutant RNA separately from the overall pathogenesis of HD (induced by mutant RNA and protein). In HD/100CAG mice, the presence of the mutant transcript itself caused a certain motor phenotype, resulted in substantial anxiety and changes in gene expression levels in the striatum. In HD/100Q mice the mutant RNA and mutant protein are expected to cause effects that partially overlap and also enhance each other. In this model, motor impairments were more significant than in HD/100CAG mice, and increased anxiety, as well as, alterations in expression levels of neuropathology‐related genes were reported.

We developed the HD/100CAG and HD/100Q mouse models, which allowed us to directly compare the effects of mutant *HTT* RNA with those of mutant *HTT* RNA and protein. We comprehensively characterized homozygous animals because the initial evaluation of heterozygous mice, at 8 months of age, showed no substantial phenotypic effects (data not shown). Overall, we identified effects common to both models, including motor impairments, increased anxiety, and gene deregulation in the striatum (but occurring with different extent and severity). A certain limitation of the HD/100Q model is the lack of typical HD molecular alterations in the striatum and late disease phenotype in this model. Presented models express transgenes with relatively moderate CAG tract mutation, as compared with other HD mouse models. However, this may have allowed some unique (but relevant to HD) features to be observed in the comparisons of the two models. We also reported some differences between HD/100CAG and HD/100Q mice in transgene expression and CAG tract mutation. Such observations have also been previously noticed in other HD mouse models.^42^, ^43^, ^44^ Nevertheless, the mentioned before differences between our models were relatively small and did not significantly affect the observed phenotype of HD/100CAG mice. One would expect that the higher average number of CAG repeats and higher expression of the transgene (Figure 2C) in the HD/100CAG model than in the HD/100Q model would cause increased toxicity of the mutant RNA. However, in the HD/100Q model, mutant RNA and mutant protein‐induced alterations in pathways likely interact to accelerate the emergence of the disease phenotype (Figure 6). A similar relationship between RNA and protein toxicity may occur in human HD.

The generated models did not show changes in body weight, whereas some HD mouse models show body weight gain or loss.^59^ The lack of change in body weight may have resulted from the relatively low level of *mHTT* expression, similar to that in BAC‐CAG mice^31^ or from a lack of full‐length mHTT, as seen in YAC128 mice, which express truncated mutant *Htt*.^60^ Nevertheless, the lack of change in body mass in our models provides a clearer interpretation of the locomotor test results, as they were not affected by the body weight of the model mice. We extensively characterized the HD/100Q and HD/100CAG models using a battery of behavioral tests, both conventional (such as the rotarod and hindlimb tests) and novel (i.e., not previously used to study HD models, such as the ActiMot system). Moreover, we characterized the model mice for a time period corresponding to almost the entire lifespan of normal WT mice.

Locomotor deficits were observed in HD/100Q mice relatively early, especially in the rotarod test (Figure 3A,B). Nevertheless, these mice generally showed slow disease progression, as few substantial changes in performance in the static rod test or gait analysis were reported at 12 months of age. Severe and rapid disease progression was clearly observed in HD/100Q mice after 18 months of age, as shown by the high phenotype scores (Figure 3G). According to the scoring system used (scoring was performed in a blinded manner), HD/100CAG mice exhibited higher scores than did WT mice, but with much milder severity than HD/100Q.

Additionally, to study some of the psychiatric symptoms reported in HD patients, we used the ActiMot system again. The system is fully automated; therefore, there is no risk of bias from a subjective experimenter. While most conventional behavioral tests are carried out in the light phase and thus reveal only some mouse behaviors, we were able to measure activity during both the light and dark phases and monitor behavior under undisturbed conditions in the 24‐h activity test. The experiments provided reference to circadian abnormalities reported in HD patients^61^ and also in HD animal models.^62^, ^63^ Our results showed increased activity in the dark phase at the 12‐ and 16‐month timepoints for both models (Figure 4A,B). This suggests that in the studied models, there were some disturbances in neuronal activation dependent on the circadian phase.^64^

Some rodent models of HD, for example, R6/2 mice^65^ and HD rats,^66^ have been shown to have reduced anxiety levels, as assessed by the elevated plus‐maze test. Nevertheless, increased anxiety is common in HD patients; it most often emerges before motor symptoms and is associated with, for example, aggression and irritability.^67^ Pharmacologic and psychological interventions for anxiety are offered to patients to improve their quality of life.^68^ In our study, anxiety‐like behavior was precisely assessed by evaluating mouse movement in nonhome cages. Both models showed increased anxiety at 12 months of age in this test (Figure 4C), whereas the HD/100CAG mice also showed anxiety at 19 months of age (Figure 4D). As the tests were performed during the light phase, the differences are expected to be even more pronounced if the tests are performed during the dark phase (due to the increased activity of mice in this phase). The lack of anxiety in HD/100Q mice at 19 months of age can be explained by the progressing locomotor phenotype. Interestingly, HD/100CAG mice showed worse performance on the static rod test at 8 and 12 months of age (Figure 3C), which could have resulted from increased anxiety. Overall, our models are suitable for studying anxiety in HD and based on the results obtained in HD/100CAG mice, *mHTT* RNA may be an important factor in inducing psychiatric symptoms such as anxiety.

We analyzed gene expression to reveal the molecular basis of phenotypic effects observed in both models. We assumed that some neuropathology‐related pathways should be deregulated which we could observe at the level of specific transcripts. In HD, mutant HTT has a progressive effect, especially in the striatum of the basal ganglia, leading to neuronal dysfunction and the death of GABAergic MSNs. Therefore, striatal gene expression has been extensively assessed in the *postmortem* brains of HD patients as well as in animal models of HD. Thus, to analyze whether the expression of genes is also altered in the striatum in our model mice expressing mutant *HTT*, we used the nCounter Mouse Neuropathology panel. It allows comprehensive analysis of the expression of genes involved in fundamental processes associated with neurodegeneration, and we identified several dozens of dysregulated genes in each of the models compared to WT mice. Among the altered pathways were those specifically involved in apoptosis, autophagy, synaptic plasticity, metabolic disturbances in neurons, and neuroinflammatory processes, which are strongly related to HD pathology,^14^ and could be related to motor disturbances observed especially in HD/100Q mice (Figure 3G). For example, altered autophagy was indicated in our models by deregulation of “chaperone‐mediated autophagy signaling” and “CLEAR signaling” pathways. On the other hand, hyperkinesis in dark phase and anxiety, clearly reported for both models (Figure 4), could be connected with deregulated various clock‐controlled metabolic genes involved in insulin signaling and mitochondrial function, for example, *Igf1r* and *Slc2a1*. Importantly, four genes were identified in both comparisons (HD/100CAG mice vs. WT mice and HD/100Q mice vs. WT mice), that showed the same direction of change in expression. Among these genes, three were downregulated (*Parp1, Igf1r*, and *Srsf4*), and one was upregulated (*Epha7*). We expect that some of the molecular changes observed in the HD/100CAG model also occur in the HD/100Q model due to the presence of the mutant transcript in both models. Therefore, due to their functions, these four genes seem to be interesting candidates for consideration of their potential involvement in RNA‐mediated HD pathogenesis.

EphA7 belongs to a large family of receptor tyrosine kinases that play essential roles during nervous system development by regulating cell migration, spatial organization, tissue patterning, axonal guidance, and establishment of synaptic connections.^69^, ^70^, ^71^, ^72^ Several studies have also demonstrated that EphA7 and its ligand ephrin‐A5 induce apoptosis in various regions in the developing brain.^73^, ^74^, ^75^ In the adult nervous system, EphA and ephrin‐A expression is largely downregulated. Still, in some family members of these regulators, expression is significantly high when developmental events occur in adulthood.^69^ For example, neuronal EphA4 cooperates with Ephrin‐A3 expressed in hippocampal glial cells to control synaptic morphology and plasticity, or ephrin‐A2, which reduces synaptic pruning in the adult cerebral cortex.^76^, ^77^ This pathway has also been linked to neuropathology, including the inhibition of regenerative processes after traumatic injury/stroke, chronic neuropathic pain, and neurodegenerative diseases.^70^ In our study, we observed significantly increased expression of the EphA7 receptor in adult mice (21 months old) expressing either the mutant transcript only or both toxic entities compared to WT mice. IPA with the core analysis function indicated Epha7 is significantly associated with canonical pathways including axonal guidance‐, ephrin A‐, ephrin receptor‐ signaling, and the synaptogenesis signaling pathway. Dysregulation of genes involved in these processes can result in abnormal apoptosis and, together with dysregulation of other genes, cause loss of striatal neurons.

The next candidate potentially involved in RNA‐related HD pathogenesis is IGF1R. We observed decreased expression of this receptor in both HD models, and it has been previously shown that disruption of IGF1/IGF1R‐associated molecular pathways may negatively affect nervous system function.^78^ Briefly, Igf1r as a part of phosphatidylinositol 3‐kinase/protein kinase B (PI3K/Akt) pathway and the mitogen‐activated protein (MAP) kinase pathway, plays important roles in neuronal development, neurogenesis, neuronal function, normal brain physiology, and metabolism.^79^, ^80^, ^81^ Several studies have demonstrated that point mutations in both IGF and IGF1R are associated with mental retardation, microcephaly, significant delays in psychomotor function, and hearing loss as a result of reduced expression of *IGF1*.^82^, ^83^, ^84^, ^85^, ^86^ A recent study by Cardoso et al.^79^ confirmed that IGF1R deficiency decreases brain weight, activates the IR/PI3K/Akt pathway, and inhibits the MAPK/ERK1/2/CREB signaling pathway. In addition to *Igf1r*, we also observed decreased expression of *Insr* and *Akt*2 in the striatum of HD/100CAG and HD/100Q mice, respectively. These results may further indicate global dysregulation of the *Igf1r* signaling pathway in our HD models, potentially leading to changes in neuronal differentiation, synapse formation, glucose metabolism, and neuronal survival. Interestingly, according to the IPA results, one of the canonical pathways inhibited in the HD/100CAG model, in which *Igf1r*, *Insr*, and other important genes were downregulated, was CREB signaling. This pathway is involved in several brain diseases, including cognitive and neurodegenerative disorders, such as HD.^87^

Among the identified downregulated genes common to the newly generated models was the splicing regulator *Srsf4*, which plays an important role in RNA metabolism by binding to both exonic and intronic sites.^88^ Moreover, SRSF4 can regulate gene expression by mediating retained intron splicing and can influence disease progression by modulating the PI3K/Akt signaling pathway.^89^ This pathway was also altered in both the HD/100Q and HD/100CAG models in our study. Dysregulation of SRSFs disrupts normal splicing, leading to the generation of pathogenic isoforms of genes and proteins, which is strongly associated with cancer progression as well as autoimmune and neurodegenerative diseases.^88^ There are no data specifically on the role of SRSF4 in HD; however, widespread dysfunction of mRNA splicing is observed in HD patients and is implicated in the development and progression of HD.^25^, ^90^ Further research is needed to verify whether SRSF4 is indeed one of the proteins responsible for the aberrant splicing observed in HD and whether this process is also associated with the presence of mutant *HTT* transcripts.

The last interesting candidate is PARP1 which generally plays a crucial role in DNA repair pathway, a process that is disrupted in multiple neurodegenerative diseases, including HD.^91^ When cells suffer massive strand breaks, PARP‐1 is excessively activated, leading to the exhaustion of NAD+ and ATP and, ultimately, cell death.^91^, ^92^, ^93^ In our studies, we observed reduced expression of *Parp1*, which contradicts most findings showing increased levels of expression of this gene in the brains of HD patients and HD mouse models.^94^, ^95^, ^96^ This result could indicate that DNA damage was not elevated in our HD models and suggest a function of Parp1 other than DNA repair in the pathogenesis of HD. Previously, it has been shown that PARP1 also plays a role in energy metabolism and neuroinflammation.^91^, ^97^ Our IPA analysis with the core analysis function indicated the involvement of Parp1 in the canonical pathways NAD signaling, RAR activation (which was inhibited), and the sirtuin signaling pathway in both models. Whereas activation of apoptotic signaling pathways was noted only in the HD/100CAG model. Interestingly, a recent study^98^ showed lower poly ADP‐ribose (PAR) levels in the CSF of HD patients even before the onset of full HD symptoms as well as reduced PARP1/2 activity in HD‐derived fibroblasts in the presence of double‐strand DNA breaks. Taken together, our results might suggest impaired PARP1 signaling which is observed in HD disease.

Insight into the contribution of mutant RNA to HD pathogenesis is important for therapeutic design. Strategies that are designed to lower the expression of *HTT* (or *mHTT* only) usually target *HTT* mRNA but do not necessarily induce transcript degradation, as lowering the protein level of HTT is usually regarded as the most crucial outcome.^99^, ^100^ Nevertheless, due to the increasing number of RNA toxicity pathways identified in HD,^23^, ^24^, ^25^ strategies that induce RNA degradation should be preferred. One of the options is also direct targeting of CAG tracts in *HTT* mRNA.^101^, ^102^, ^103^ Other approaches that abolish mutant RNA toxicity (in addition to mutant protein toxicity) include gene editing strategies, which were recently tested in preclinical studies of HD.^104^, ^105^, ^106^ An increased understanding of the role of *HTT* transcripts as pathogenic agents, together with the improvement of strategies that target these transcripts, should lead to the successful development of therapies for HD.

## 5 CONCLUSION

HD has an extremely complex pathology in which multiple mechanisms may occur simultaneously and contribute to disease development and progression. In simple terms, the abnormalities observed in the HD/100CAG model, which are due to the presence of the mutant transcript, should also occur in the HD/100Q model. Nevertheless, the molecular changes in the HD/100Q model are more complex than those in the HD/100CAG model due to the coexistence of both the mutant transcript and the mutant protein (Figure 6).

According to the results obtained from HD/100CAG and HD/100Q models, the mutant protein is expected to contribute more strongly to HD pathogenesis than the mutant transcript. However, RNA and protein toxicity may impact and intensify each other in HD. Therefore, it can be assumed that mutant RNA‐related effects are less prominent in HD/100 CAG mice than in HD/100Q mice (Figure 6). In HD/100Q mice we observed late locomotor phenotype and gene expression alterations related to pathways typically affected in HD. Although generally this model is limited in reflecting pathological abnormalities associated with HD, it was created as a reference model to investigate alterations in HD/100CAG mice. Using the HD/100CAG model, we showed that the presence of the mutant transcript itself might cause psychiatric symptoms such as anxiety and lead to changes in gene expression, which could be related to common hallmarks of HD.^19^, ^107^, ^108^ Nevertheless, no clear disease phenotype could be observed in this model. Therefore, our results suggest a possible but not critical contribution of disruptions caused by mutant RNA to the pathogenesis of HD. Based on the observed phenotype in HD/100CAG mice and its comparison to HD/100Q mice, we conclude that *mHTT* transcript itself may have pathological implications for the development of hyperkinesis and anxiety in HD. We provide some indications of genes with deregulated expression for a molecular understanding of phenotypical alterations but additional experiments should address the precise mechanisms. Potentially, a transcriptomic analysis that is less biased toward gene composition, encompasses both presymptomatic and symptomatic timepoints, and includes additional brain regions, would provide a more comprehensive means to establish a mechanistic link between changes in gene expression patterns and the locomotor deficits observed. Further research is also needed to address the issue of the ability of mutant RNA to participate in disrupted pathways leading directly to neurodegeneration. Investigation of mutant RNA in vivo localization, interactions, and metabolism, will enable the identification of direct implications of *HTT* transcript in HD pathology.

## AUTHOR CONTRIBUTIONS

Coordination of the study: Agnieszka Fiszer. Mouse models design: Pawel Michal Switonski. Research design: Magdalena Jazurek‐Ciesiolka, Lukasz Przybyl, Magdalena Wozna‐Wysocka, Pawel Michal Switonski, Agnieszka Fiszer, Joanna Suszynska‐Zajczyk. Performing molecular experiments: Magdalena Wozna‐Wysocka, Magdalena Jazurek‐Ciesiolka, Pawel Michal Switonski, Julia Oliwia Misiorek, Grzegorz Figura, Paula Sobieszczanska, Lukasz Przybyl. Performing behavioral experiments: Lukasz Przybyl, Magdalena Wozna‐Wysocka, Dorota Wronka, Joanna Suszynska‐Zajczyk. Mice sections: Lukasz Przybyl, Dorota Wronka, Magdalena Wozna‐Wysocka, Pawel Michal Switonski, Julia Oliwia Misiorek. Performing nCounter analysis: Anna Zeller, Magdalena Niemira. Analysis of the data: Magdalena Wozna‐Wysocka, Lukasz Przybyl, Magdalena Jazurek‐Ciesiolka, Agnieszka Fiszer, Pawel Michal Switonski, Dorota Wronka, Julia Oliwia Misiorek, Grzegorz Figura, Adam Ciesiolka. Funding acquisition: Adam Ciesiolka, Agnieszka Fiszer, Magdalena Jazurek‐Ciesiolka, Joanna Suszynska‐Zajczyk. Figures preparation: Magdalena Wozna‐Wysocka, with contributions from Julia Oliwia Misiorek, Lukasz Przybyl, Dorota Wronka, Magdalena Jazurek‐Ciesiolka, and Agnieszka Fiszer. Manuscript writing: Magdalena Wozna‐Wysocka, Agnieszka Fiszer, Magdalena Jazurek‐Ciesiolka, Lukasz Przybyl, and Pawel Michal Switonski with input and revision from all the authors. All the authors approved the final version of the manuscript.

## FUNDING INFORMATION

This study was supported by grants from the National Science Centre, Poland [2012/06/A/NZ1/00094—generation of the models, initial molecular and motor characteristics, establishment of the cohort; 2015/19/B/NZ2/02453—molecular characteristics of cohort by RT‐qPCR and western blot, behavioral tests; 2015/19/D/NZ5/02183—immunohistochemistry and immunoprecipitation analyses; 2016/21/D/NZ4/00478—behavioral tests; 2021/41/B/NZ3/03803—smFISH and molecular characteristics of cohort by Neuropathology Panel].

## DISCLOSURES

The authors declare that they have no competing interests.

## Supporting information


Text S1.
Text S2.
Text S3.
Table S1.

Figure S1.

Figure S2.

Figure S3.

Figure S4.

Figure S5.

Figure S6.

Figure S7.

Data S1.

## Data Availability

The nCounter data are openly available in NCBI's Gene Expression Omnibus^109^ and are accessible through GEO Series accession number GSE269068 (https://www.ncbi.nlm.nih.gov/geo/query/acc.cgi?acc=GSE269068). Other datasets analyzed during the current study are included in the supplementary files.
