# Supplementary material for: Insights into RNA‐mediated pathology in new mouse models of Huntington's disease
Source: FASEB J. 2024 Nov 27;38(23):e70182. doi: 10.1096/fj.202401465R (PMC11602643; doi:10.1096/fj.202401465R)
Supplement: Supplementary file 1 — Text S1. Text S2. Text S3. Table S1. [file FSB2-38-e70182-s008.docx]

**Supplementary Texts, Table and Figures legends**

Supplementary information content list:

**Text S1**. Nucleotide and amino acid sequences of *HTT* transgene.

**Text S2**. Description of locus *Rosa*26 modification.

**Text S3.** Description of Supplementary file 1 (Excel file).

**Table S1.** Sequence of Primers utilized for PCR and RT-qPCR.

**Supplementary Figures legends**

**Text S1.** Nucleotide and amino acid sequences of *HTT* transgene. Start and stop codons are shown in red letters.

**HD/100Q DNA:**

CTCGAG**ATG**GCGACCCTGGAAAAGCTG**ATG**AAGGCCTTCGAGTCCCTCAAGTCCTTCCAGCAGCAGCAGCAGCAGCAGCAGCAGCAGCAGCAGCAGCAGCAGCAGCAGCAGCAGCAGCAGCAGCAGCAGCAGCAGCAGCAGCAGCAGCAGCAGCAGCAGCAGCAGCAGCAGCAGCAGCAGCAGCAGCAGCAGCAGCAGCAGCAGCAGCAGCAGCAGCAGCAGCAGCAGCAGCAGCAGCAGCAGCAGCAGCAGCAGCAGCAGCAGCAGCAGCAGCAGCAGCAGCAGCAGCAGCAGCAGCAGCAGCAGCAGCAGCAGCAGCAGCAGCAGCAGCAGCAGCAGCAGCAGCAGCAGCAACAGCCGCCACCGCCGCCGCCGCCGCCGCCGCCTCCTCAGCTTCCTCAGCCGCCGCCGCAGGCACAGCCGCTGCTGCCTCAGCCGCAGCCGCCCCCGCCGCCGCCCCCGCCGCCACCCGGCCCGGCTGTGGCTGAGGAGCCGCTGCACCGACCAAAGAAAGAACTTTCAGCTACCAAGAAAGACCGTGTGAATCATTGTCTGACAATATGTGAAAACATAGTGGCACAGTCTGTCAGAAATTCTCCAGAATTTCAGAAACTTCTGGGCATCGCTATGGAACTTTTTCTGCTGTGCAGTGATGACGCAGAGTCAGATGTCAGGATGGTGGCTGACGAATGCCTCAACAAAGTTATCAAAGCTTTGATGGATTCTAATCTTCCAAGGTTACAGCTCGAATACCCATACGATGTTCCAGATTACGCT**TGA**CCTGCATGGGAGCTTAGTTGGATCACCTAGAACAGCAAGATCTGAGGATCCTAAGGTACCTAATTGCCTAGAAAACATGAGGATCACCCATGTCTGCAGGTCGACTCTAGAAAACATGAGGATCACCCATGTCTGCAGTATTCCCGGGTTCATTAGATCCTAAGGTACCTAATTGCCTAGAAAACATGAGGATCACCCATGTCTGCAGGTCGACTCTAGAAAACATGAGGATCACCCATGTCTGCAGTATTCCCGGGTTCATTAGATCCTAAGGTACCTAATTGCCTAGAAAACATGAGGATCACCCATGTCTGCAGGTCGACTCCAGAAAACATGAGGATCACCCATGTCTGCAGTATTCCCGGGTTCATTAGATCCTAAGGTACCTAATTGCCTAGAAAACATGAGGATCACCCATGTCTGCAGGTCGACTCTAGAAAACATGAGGATCACCCATGTCTGCAGTATTCCCGGGTTCATTAGATCCTAAGGTACCTAATTGCCTAGAAAACATGAGGATCACCCATGTCTGCAGGTCGACTCTAGAAAACATGAGGATCACCCATGTCTGCAGTATTCCCGGGTTCATTAGATCCTAAGGTACCTAATTGCCTAGAAAACATGAGGATCACCCATGTCTGCAGGTCGACTCCAGAAAACATGAGGATCACCCATGTCTGCAGTATTCCCGGGTTCATTAGATCCTAAGGTACCTAATTGCCTAGAAAACATGAGGATCACCCATGTCTGCAGGTCGACTCTAGAAAACATGAGGATCACCCATGTCTGCAGTATTCCCGGGTTCATTAGATCCTAAGGTACCTAATTGCCTAGAAAACATGAGGATCACCCATGTCTGCAGGTCGACTCTAGAAAACATGAGAGGATCACCCATGTCTGCAGTATTCCCGGGTTCATTAGATCCTAAGGTACCTAATTGCCTAGAAAACATGAGGATCACCCATGTCTGCAGGTCGACTCTAGAAAACATGAGGATCACCCATGTCTGCAGTATTCCCGGGTTCATTAGATCCTAAGGTACCTAATTGCCTAGAAAACATGAGGATCACCCATGTCTGCAGGTCGACTCTAGAAAACATGAGGATCACCCATGTCTGCAGTATTCCCGGGTTCATTAGATCCTAAGGTACCTAATTGCCTAGAAAACATGAGGATCACCCATGTCTGCAGGTCGACTCTAGAAAACATGAGGATCACCCATGTCTGCAGTATTCCCGGGTTCATTAGATCCTAAGGTACCTAATTGCCTAGAAAACATGAGGATCACCCATGTCTGCAGGTCGGACTCTAGAAAACATGAGGATCACCCATGTCTGCAGTATTCCCGGGTTCATTAGATCTGCCGCGCGATCGATATGTAGCGGCCGC

XhoI HTT HA-tag Linker BamHI MS2 ClaI NotI

CAG repeats

**HD/100Q protein:**

MATLEKLMKAFESLKSFQQQQQQQQQQQQQQQQQQQQQQQQQQQQQQQQQQQQQQQQQQQQQQQQQQQQQQQQQQQQQQQQQQQQQQQQQQQQQQQQQQQQQQQQQQQQQQQQQQQQPPPPPPPPPPPQLPQPPPQAQPLLPQPQPPPPPPPPPPGPAVAEEPLHRPKKELSATKKDRVNHCLTICENIVAQSVRNSPEFQKLLGIAMELFLLCSDDAESDVRMVADECLNKVIKALMDSNLPRLQLEYPYDVPDYA-

**HD/100CAG DNA:**

CTCGAG**TGA**GCGACCCTGGAAAAGCTG**TGA**AAGGCCTTCGAGTCCCTCAAGTCCTTCCAGCAGCAGCAGCAGCAGCAGCAGCAGCAGCAGCAGCAGCAGCAGCAGCAGCAGCAGCAGCAGCAGCAGCAGCAGCAGCAGCAGCAGCAGCAGCAGCAGCAGCAGCAGCAGCAGCAGCAGCAGCAGCAGCAGCAGCAGCAGCAGCAGCAGCAGCAGCAGCAGCAGCAGCAGCAGCAGCAGCAGCAGCAGCAGCAGCAGCAGCAGCAGCAGCAGCAGCAGCAGCAGCAGCAGCAGCAGCAGCAGCAGCAGCAGCAGCAGCAGCAGCAGCAGCAGCAGCAGCAGCAGCAGCAGCAGCAACAGCCGCCACCGCCGCCGCCGCCGCCGCCGCCTCCTCAGCTTCCTCAGCCGCCGCCGCAGGCACAGCCGCTGCTGCCTCAGCCGCAGCCGCCCCCGCCGCCGCCCCCGCCGCCACCCGGCCCGGCTGTGGCTGAGGAGCCGCTGCACCGACCAAAGAAAGAACTTTCAGCTACCAAGAAAGACCGTGTGAATCATTGTCTGACAATATGTGAAAACATAGTGGCACAGTCTGTCAGAAATTCTCCAGAATTTCAGAAACTTCTGGGCATCGCTATGGAACTTTTTCTGCTGTGCAGTGATGACGCAGAGTCAGATGTCAGGATGGTGGCTGACGAATGCCTCAACAAAGTTATCAAAGCTTTGATGGATTCTAATCTTCCAAGGTTACAGCTCGAATACCCATACGATGTTCCAGATTACGCT**TGA**CCTGCATGGGAGCTTAGTTGGATCACCTAGAACAGCAAGATCTGAGGATCCTAAGGTACCTAATTGCCTAGAAAACATGAGGATCACCCATGTCTGCAGGTCGACTCTAGAAAACATGAGGATCACCCATGTCTGCAGTATTCCCGGGTTCATTAGATCCTAAGGTACCTAATTGCCTAGAAAACATGAGGATCACCCATGTCTGCAGGTCGACTCTAGAAAACATGAGGATCACCCATGTCTGCAGTATTCCCGGGTTCATTAGATCCTAAGGTACCTAATTGCCTAGAAAACATGAGGATCACCCATGTCTGCAGGTCGACTCCAGAAAACATGAGGATCACCCATGTCTGCAGTATTCCCGGGTTCATTAGATCCTAAGGTACCTAATTGCCTAGAAAACATGAGGATCACCCATGTCTGCAGGTCGACTCTAGAAAACATGAGGATCACCCATGTCTGCAGTATTCCCGGGTTCATTAGATCCTAAGGTACCTAATTGCCTAGAAAACATGAGGATCACCCATGTCTGCAGGTCGACTCTAGAAAACATGAGGATCACCCATGTCTGCAGTATTCCCGGGTTCATTAGATCCTAAGGTACCTAATTGCCTAGAAAACATGAGGATCACCCATGTCTGCAGGTCGACTCCAGAAAACATGAGGATCACCCATGTCTGCAGTATTCCCGGGTTCATTAGATCCTAAGGTACCTAATTGCCTAGAAAACATGAGGATCACCCATGTCTGCAGGTCGACTCTAGAAAACATGAGGATCACCCATGTCTGCAGTATTCCCGGGTTCATTAGATCCTAAGGTACCTAATTGCCTAGAAAACATGAGGATCACCCATGTCTGCAGGTCGACTCTAGAAAACATGAGAGGATCACCCATGTCTGCAGTATTCCCGGGTTCATTAGATCCTAAGGTACCTAATTGCCTAGAAAACATGAGGATCACCCATGTCTGCAGGTCGACTCTAGAAAACATGAGGATCACCCATGTCTGCAGTATTCCCGGGTTCATTAGATCCTAAGGTACCTAATTGCCTAGAAAACATGAGGATCACCCATGTCTGCAGGTCGACTCTAGAAAACATGAGGATCACCCATGTCTGCAGTATTCCCGGGTTCATTAGATCCTAAGGTACCTAATTGCCTAGAAAACATGAGGATCACCCATGTCTGCAGGTCGACTCTAGAAAACATGAGGATCACCCATGTCTGCAGTATTCCCGGGTTCATTAGATCCTAAGGTACCTAATTGCCTAGAAAACATGAGGATCACCCATGTCTGCAGGTCGGACTCTAGAAAACATGAGGATCACCCATGTCTGCAGTATTCCCGGGTTCATTAGATCTGCCGCGCGATCGATATGTAGCGGCCGC

XhoI HTT HA-tag Linker BamHI MS2 ClaI NotI

CAG repeats

**Text S2.** Description of locus *Rosa*26 modification.

To modify the *Rosa26* locus, the targeting vector (containing short, ~1.1 kb, and long, ~4.3 kb, homology arms and HTT transgene, Text S1) was linearized and electroporated into C57Bl/6-derived embryonic stem cells (ESCs). For both HD/100Q and HD/100CAG lines, ESC clones were isolated and screened for homologous recombination with Southern blot and long-range PCR on the short and long homology arms, respectively (Fig. S1a,b). Clones without the MS2 aptamer and/or with a CAG repeat region that was too short as determined by digesting the 7.1 kb long-range PCR amplicon and performing fragment length analysis (Fig. S1c) were excluded. Ultimately, the occurrence of homologous recombination within the Rosa26 locus in the selected ESC clones was validated through Southern blot analysis using two probes located outside of the homology arms (Fig. S1d).

**Text S3.** Description of supplementary file 3 (Excel file).

Supplementary file 1 (Excel file) – this file contains full list of ingenuity canonical pathways (IPA, QIAGEN) for significantly deregulated genes (*p*≤0.05) obtained from NanoString analysis.

Sheet 1 – complete list of ingenuity canonical pathways for HD/100Q model, significantly altered >1.3 [–log(p-value)]

Sheet 2 – complete list of ingenuity canonical pathways for HD/100CAG model, significantly altered >1.3 [–log(p-value)]

Sheet 3 – significantly altered ingenuity canonical pathways common to both models

Sheet 4 – legend for IPA analysis (sheets 1-3).

**Supplementary Table:**

**Table S1.** Sequence of primers utilized for PCR and RT-qPCR.

| Mouse Gene/Function | 5’-3’ Primer Sequence (Forward) | 5’-3’ Primer Sequence (Reverse) | Product Length (bp) |
| --- | --- | --- | --- |
| *Clonal selection* |  |  |  |
| Rosa_forward/ Rosa_reverse Rosa26 probe generation | ACACTTATTGGCCGGTGCGCCGCCAATC | AATGGGCTGACCGCTTCCTCGTGCTTT | 608 |
| C011.5/ E573.3  Confirmation of recombination with long-range PCR | ATGTGGTGCAGTGTTGAGGGCAATCTGG | GGCGCGCCCTCGAGATGGCGACCCTGGAAAAG | 7100 |
| RO26.6/ RO26.7  Southern blot probe generation | GTGGCTTGACTTGTCACTG | GATGGCTGGCATCTTCTG | 530 |
| CAG.1/ LRPCRneo1  CAG promoter screening | TTCGGCTTCTGGCGTGTG | AATGGGCTGACCGCTTCCTCGTGCTTT | 407 |
| *Genotyping:* |  |  |  |
| The presence of CAG repeats | TCGGCGCGCACCGTACGTCTCGAG | TCGGTGCAGCGGCTCCTCAGCCAC | 522 |
| Distinguishing heterozygotes from homozygotes for the transgene | CCTCGTGATCTGCAACTCCA | GAGTCCTGGGTGTAAGTGCC  ATGCTCTGTCTAGGGGTTGG | 329  521 |
| The presence of the Cre-recombinase gene | TCCATGAGTGAACGAACCTGGTCG | GCTGTTCTCCTCTTCCTCATCTCC | 500 |
| The presence of STOP cassette | TTCGGCTTCTGGCGTGTG | AATGGGCTGACCGCTTCCTCGTGCTTT | 407 |
| The deletion of the STOP cassette | TTCGGCTTCTGGCGTGTG | GCTGCTGGAAGGACTTGAGG | 329 |
| *Capillary electrophoresis:* | 6-Fam-GCGACCCTGGAAAAGCTGATGA | Joe-CGGCGGTGGCGGCTGTTG | 360 (98 CAG) |
| *Striatal and cortical gene expression:* | | | |
| *HTT*  Fragment of human huntingtin - transgene | GCTGACGAATGCCTCAACAAAG | AACTAAGCTCCCATGCAGGTC | 118 |
| *Htt*  endogenous mouse huntingtin | AAGAAGGAACTCTCAGCCACCA | CTGAGAGACTGTGCCACAATGTT | 83 |
| *Atp5b*  ATP synthase F1 subunit beta | ACCTCGGTGCAGGCTATCTA | AATAGCCCGGGACAACACAG | 105 |
| *Gapdh*  Glyceraldehyde-3-phosphate dehydrogenase | TGTGTCCGTCGTGGATCTGA | CCTGCTTCACCACCTTCTTGA | 77 |

**Supplementary Figures Legends**

**Figure S1.** Clone selection analysis. **a** Screening of ESC clones *via* Southern blot analysis. Transgene insertion (named TG Rosa) elongated the sequence and created additional BglII restriction sites, changing the fragment recognized by the 5’ external probe, as compared to WT Rosa. **b** Long-range PCR analysis of correct recombination on the long arm of homology. Primers indicated by arrows generate a 7.1 kb-long product. DNA from a WT mouse was used as a control. **c** Digestion of fragments from the long-range PCR analysis. 7.1 kb PCR fragments were digested with HindIII. The expected digestion pattern of the intact transgene is depicted on the left. The gel shows correct clones containing the complete CAG repeat and the MS2 aptamer region highlighted by red ellipses. **d** Southern blot confirmation of homologous recombination. DNA from positive ESC clones was digested using the restriction enzymes BclI and ApaI, respectively. Correct homologous recombination to the *Rosa*26 locus changed the restriction fragments recognized with the 3’ external probe (blue) and a 5’ external probe (red). DNA from a wild-type mouse was used as a control.

**Figure S2.** Analysis of transcript levels of *HTT* transgene and endogenous *Htt* in brain regions from generated HD mouse models using the RT-qPCR in samples isolated at 4, 8, 12 and 21 months of age. **a** Quantification of *HTT* transgene expression in cortex and hippocampus from HD/100Q and HD/100CAG models. **b** Comparison of delta Ct values obtained from RT-qPCR for analysis of the expression of *HTT* transgene and mouse *Htt* in the striatum from HD/100Q model. Delta Ct values were calculated as the difference of the Ct value for *HTT* transgene/*Htt* and the mean Ct value for *Atp5b* and *Gapdh.* **c** Expression analysis of the endogenous mouse *Htt* in the striatum, cortex and hippocampus from HD/100Q, HD/100CAG and WT littermates.

*Atp5b* and *Gapdh* were used as reference genes. The number of animals per group is shown in Fig. 1. Statistical analysis has been performed using two-way ANOVA followed by Bonferroni’s; *****p* < 0.0001, (*HD/100Q mice vs. HD/100CAG mice) (a) or Tukey’s (c) *post hoc* test; *p<0.05, ## *p*<0,001; (# HD/100Q mice vs. WT mice, * HD/100CAG mice vs WT mice). The data are presented as mean ± SEM, normalized to the 4-month-old HD/100Q (a) and WT(c) mice.

**Figure S3.** Immunoblot from input and immunoprecipitated samples from HD/100Q (left panel) and HD/100CAG (right panel) mice, with samples from WT mice. Immunoprecipitation was performed on whole cortical lysates, from 21-month-old  mice, using anti-HA-tag agarose. The membranes were probed with anti-HA-tag antibody. Bands corresponding to mutant HTT fragments are indicated by red arrows. Fragments of Ponceau S-stained membranes are shown as used for verification of consistency in sample preparation.

**Figure S4.** Additional images of smFISH for mutant *HTT* RNA in MEFs isolated from E16.5 HD/100CAG mouse embryos and negative control MEFs isolated from WT mice. The white triangles indicate exemplary specific spots of *HTT* RNA. Nuclei were stained with DAPI. Scale bar = 10 µm.

**Figure S5.** Results of body and organ weight reported from 4 to 21 months of age for WT, HD/100CAG and HD/100Q mice. **a** Summary of body mass. **b**, **c**, **d**, **e** Weights of organs including brain (b), heart (c), kidney (d) and spleen (e). The number of animals per group is shown in Fig. 1. The statistical results presented in the figure legend were obtained by two-way ANOVA (main effect of genotype) followed by Tukey’s test; while #, *, ^ indicate a simple effect within rows according to Tukey’s test (# HD/100Q mice vs. WT mice; * HD/100CAG mice vs. WT mice; ^ HD/100Q mice vs. HD/100CAG mice). *p<0.05; **p<0.01; ***p<0.001; ****p<0.0001 (the same ranges also apply for ^ and #).

**Figure S6.** Serum biochemistry analysis of mice at 12 months of age. Data shown as the mean ± SEM serum concentrations of alkaline phosphatase, alanine aminotransferase, cholesterol and uric acid. One-way ANOVA was used for statistical analysis, followed by Tukey’s test. n = 6

**Figure S7.** Immunohistochemistry of the striatum and cortex of 12-month-old WT, HD/100Q and HD/100CAG mice using HD-polyAla-Ct and HD-polySer-Ct antibodies. Scale bar: 50 μm. n = 2
