# Supplementary figures and images for: Insights into RNA‐mediated pathology in new mouse models of Huntington's disease
Source: FASEB J. 2024 Nov 27;38(23):e70182. doi: 10.1096/fj.202401465R (PMC11602643; doi:10.1096/fj.202401465R)

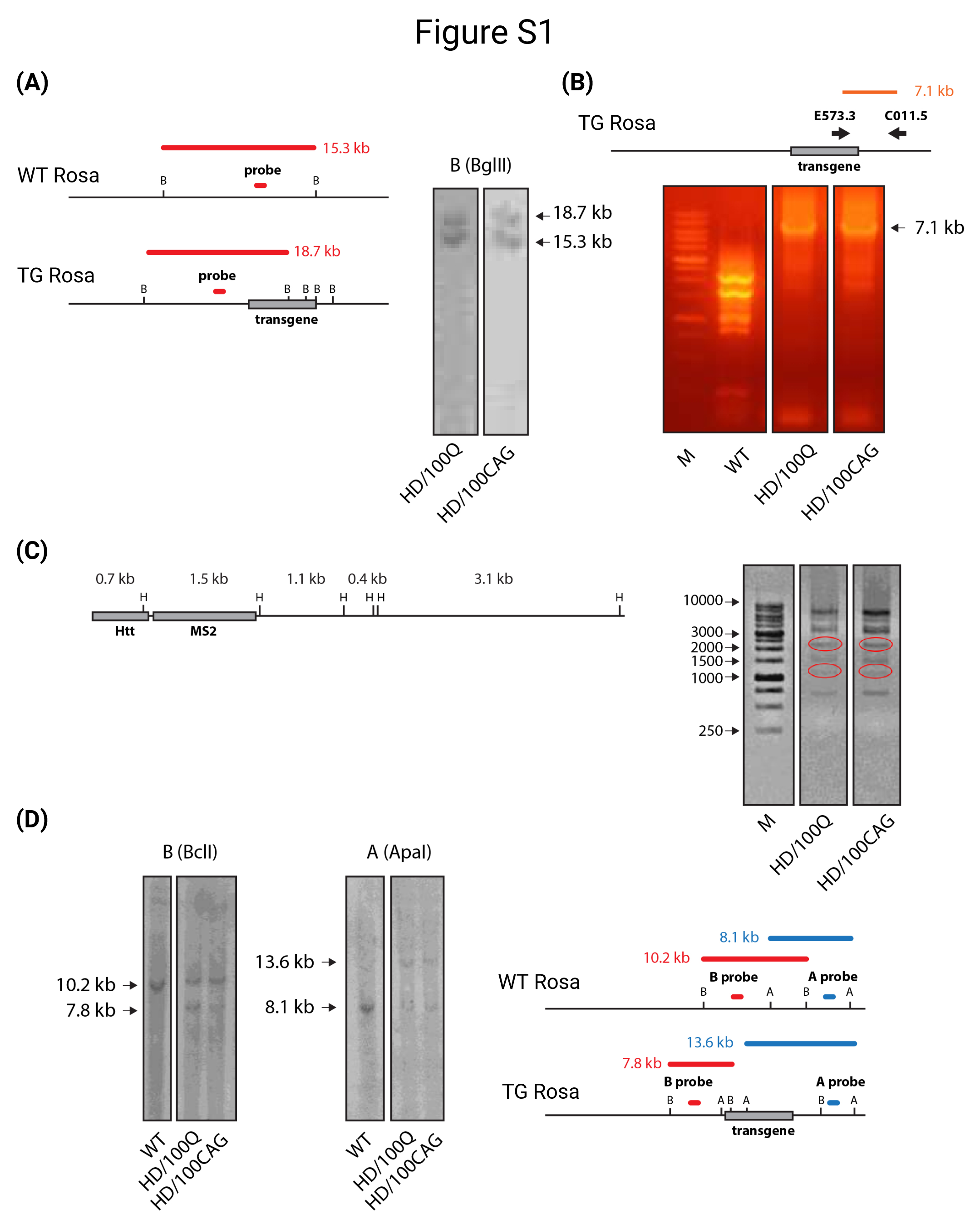

Supplement: Supplementary file 2 — Figure S1. [file FSB2-38-e70182-s009.png]

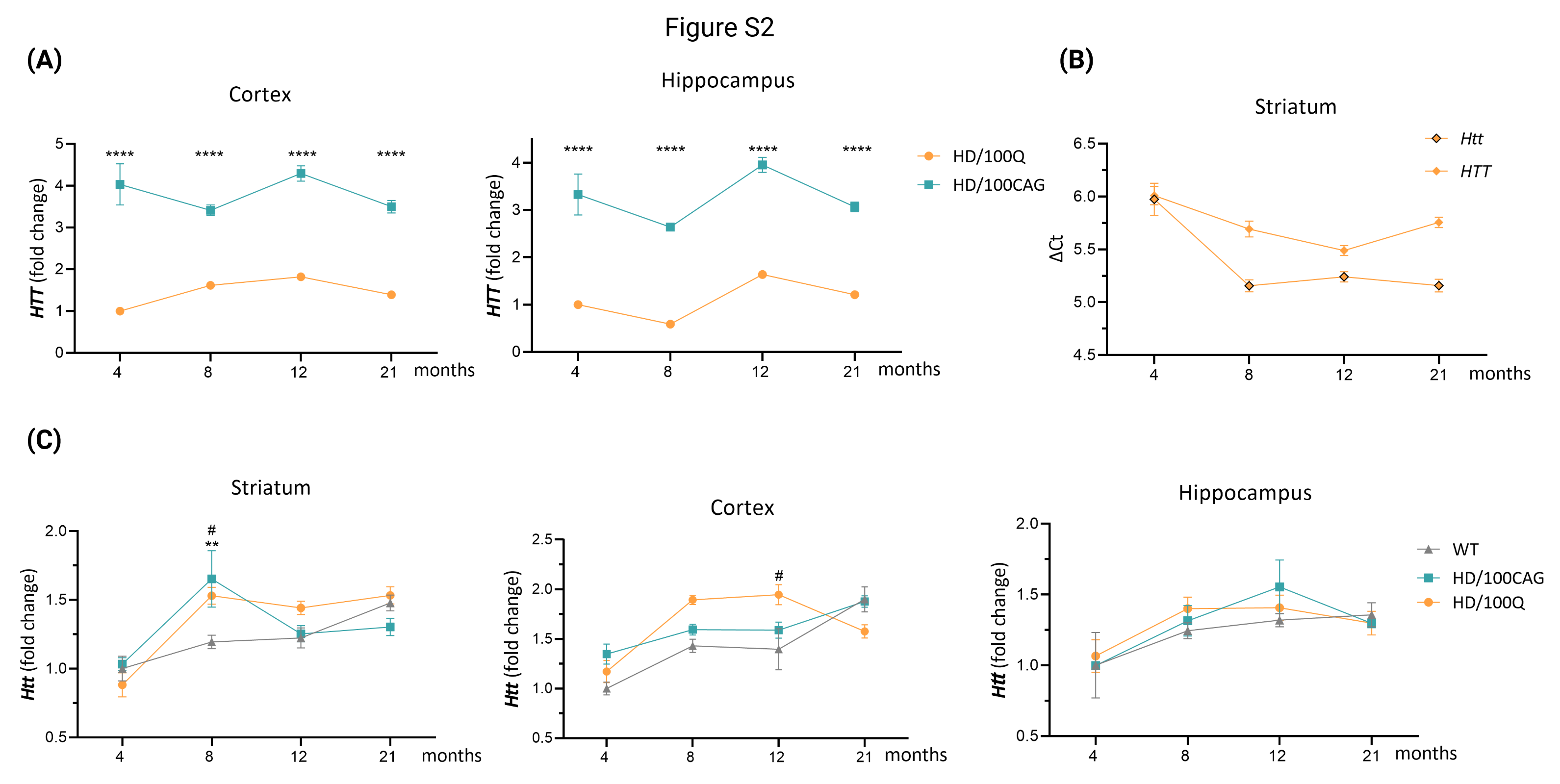

Supplement: Supplementary file 3 — Figure S2. [file FSB2-38-e70182-s006.png]

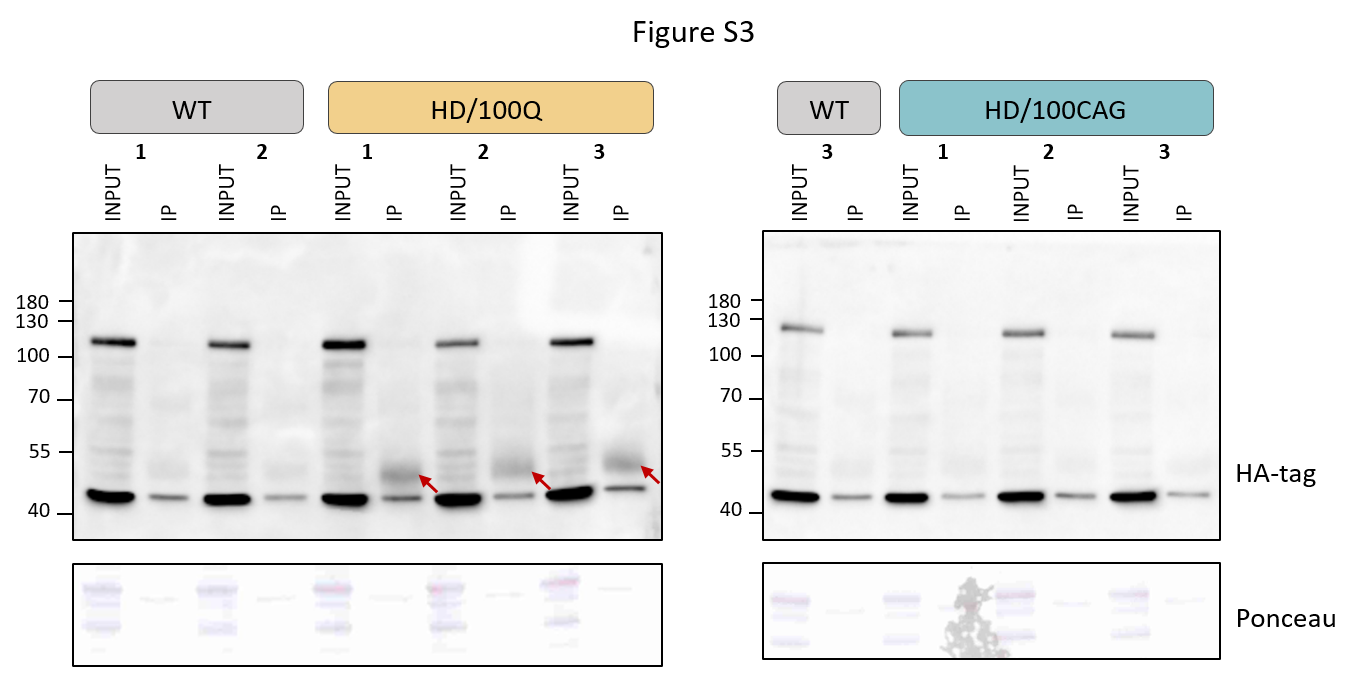

Supplement: Supplementary file 4 — Figure S3. [file FSB2-38-e70182-s004.png]

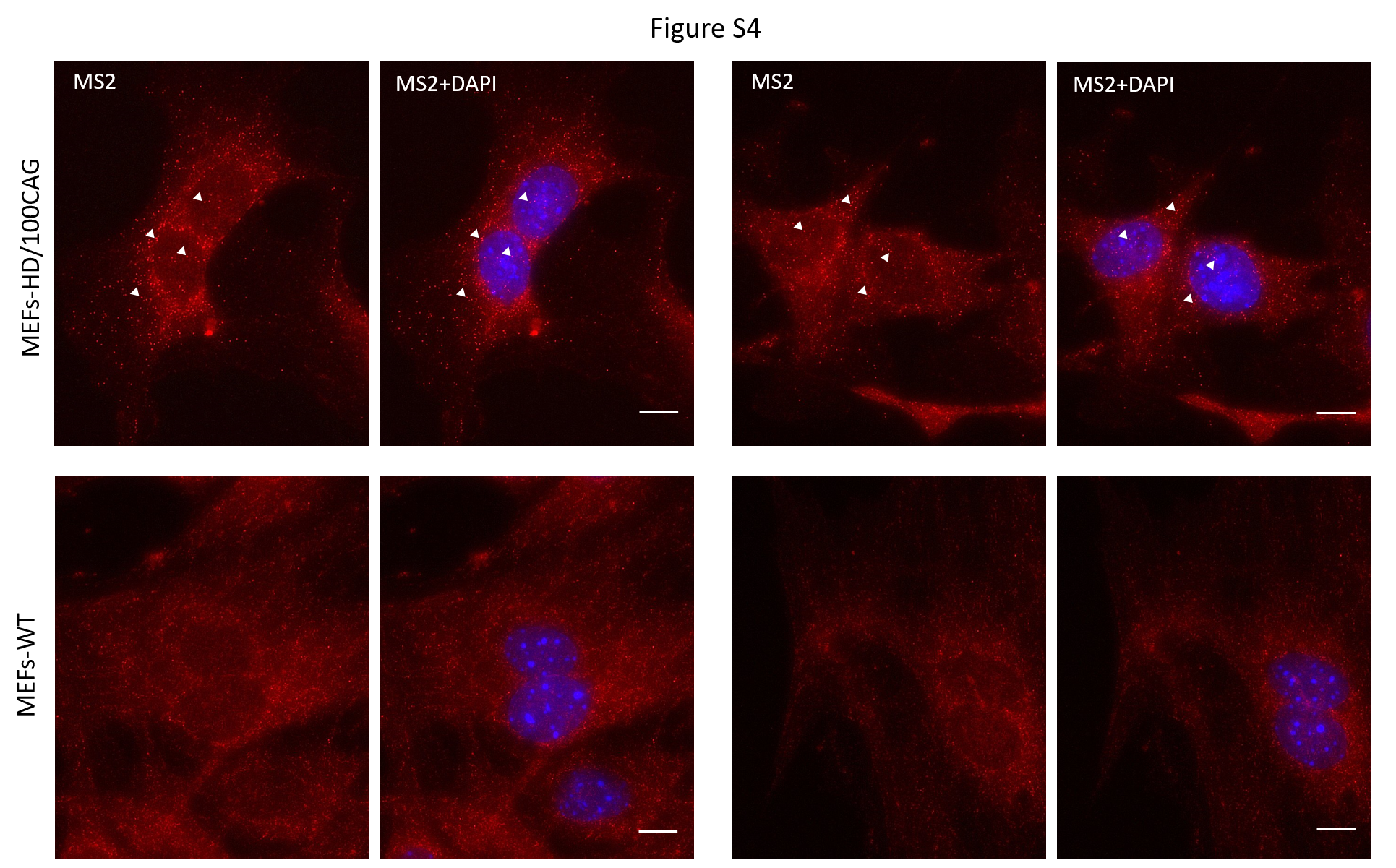

Supplement: Supplementary file 5 — Figure S4. [file FSB2-38-e70182-s007.png]

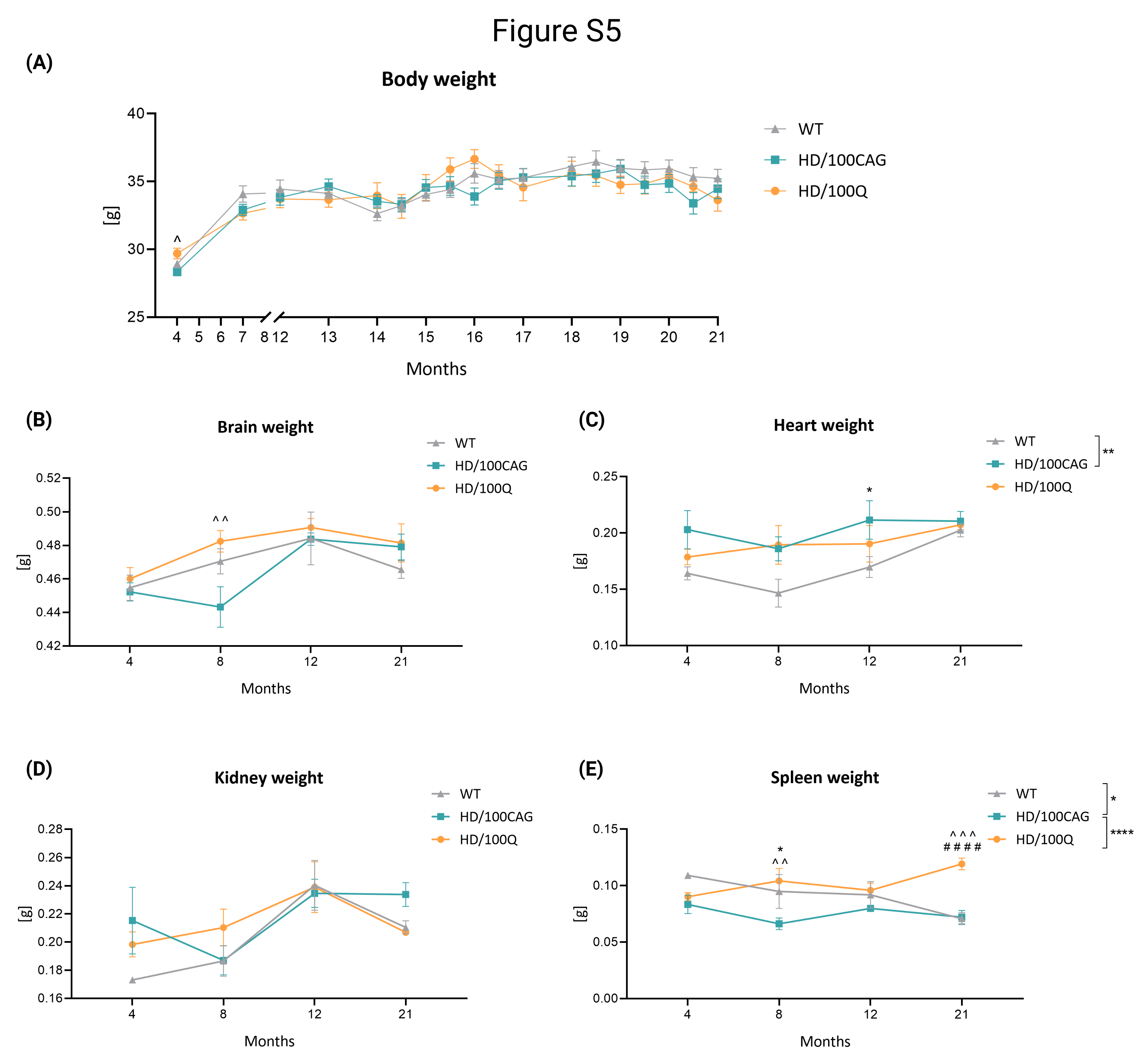

Supplement: Supplementary file 6 — Figure S5. [file FSB2-38-e70182-s005.png]

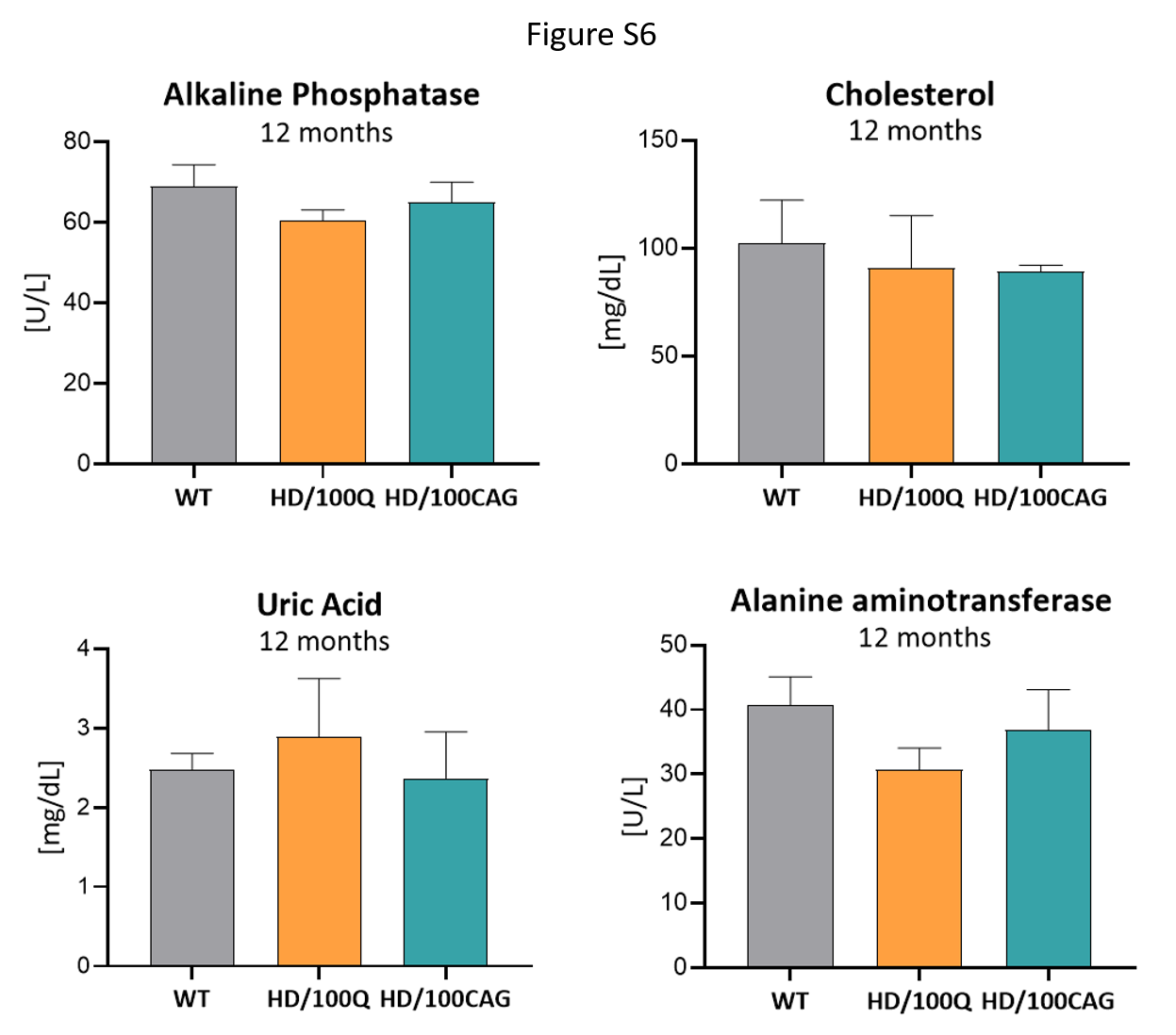

Supplement: Supplementary file 7 — Figure S6. [file FSB2-38-e70182-s003.png]

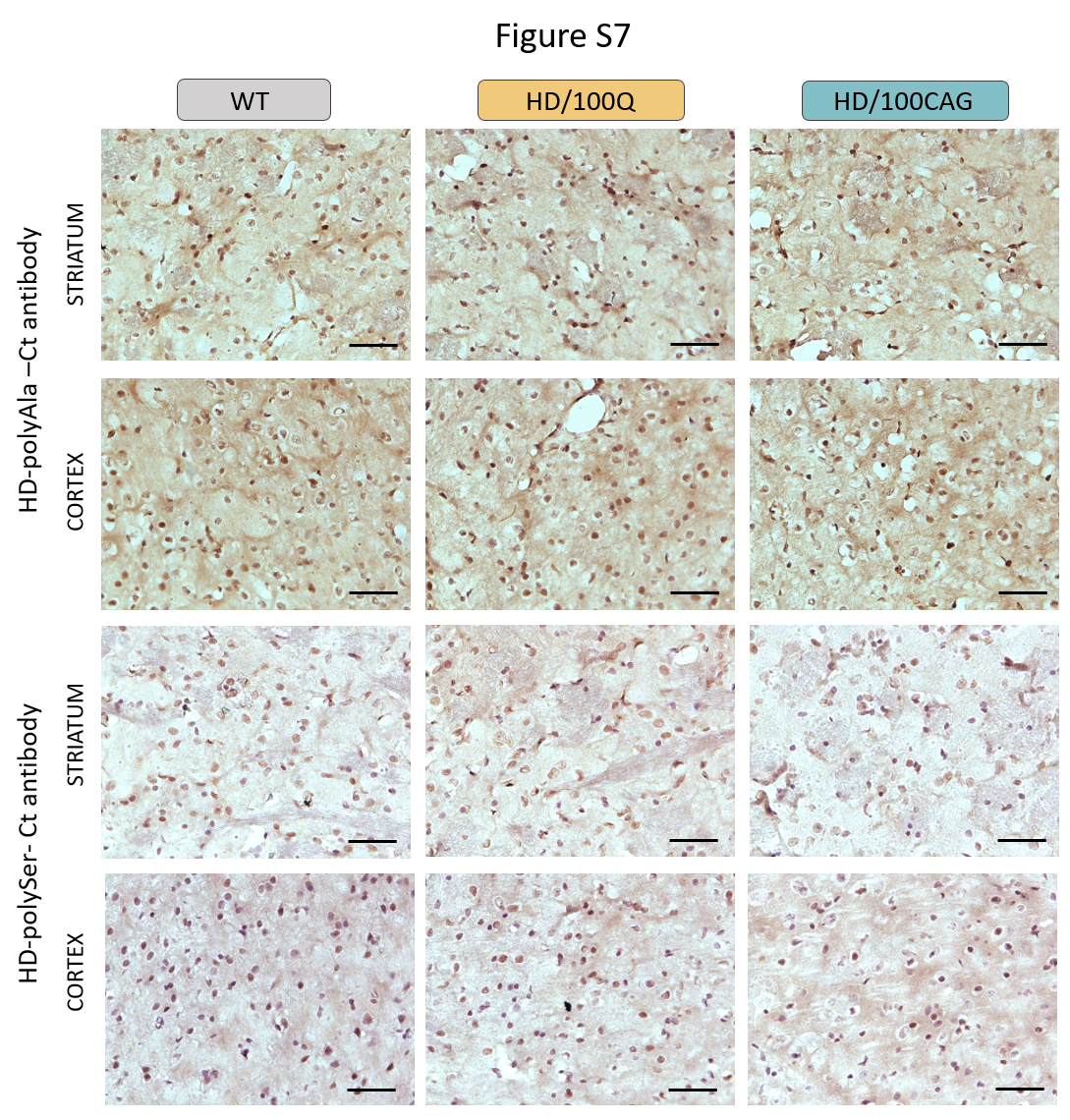

Supplement: Supplementary file 8 — Figure S7. [file FSB2-38-e70182-s002.png]
